# Clearing Up Discrepancies in 2D and 3D Nickel Molybdate Hydrate Structures

**DOI:** 10.1021/acs.inorgchem.3c03261

**Published:** 2024-01-19

**Authors:** Robin N. Dürr, Pierfrancesco Maltoni, Shihui Feng, Sagar Ghorai, Petter Ström, Cheuk-Wai Tai, Rafael B. Araujo, Tomas Edvinsson

**Affiliations:** † Department of Chemistry, Physical Chemistry, Ångström Laboratory, Uppsala University, Uppsala 751 20 ,Sweden; ‡ Université Paris-Saclay, CEA, CNRS, NIMBE, LICSEN, Gif-sur-Yvette91191 ,France; § Department of Materials Science and Engineering, Solid State Physics, Ångström Laboratory, Uppsala University, Uppsala751 03 ,Sweden; ∥ Department of Materials and Environmental Chemistry, Stockholm University, Stockholm 106 91 ,Sweden; ⊥ Department of Physics and Astronomy, Applied Nuclear Physics, Ångström Laboratory, Uppsala University, Uppsala751 20 ,Sweden; # Energy Materials Laboratory, Chemistry: School of Natural and Environmental Science, Newcastle University, Newcastle upon Tyne NE1 7RU, United Kingdom

## Abstract

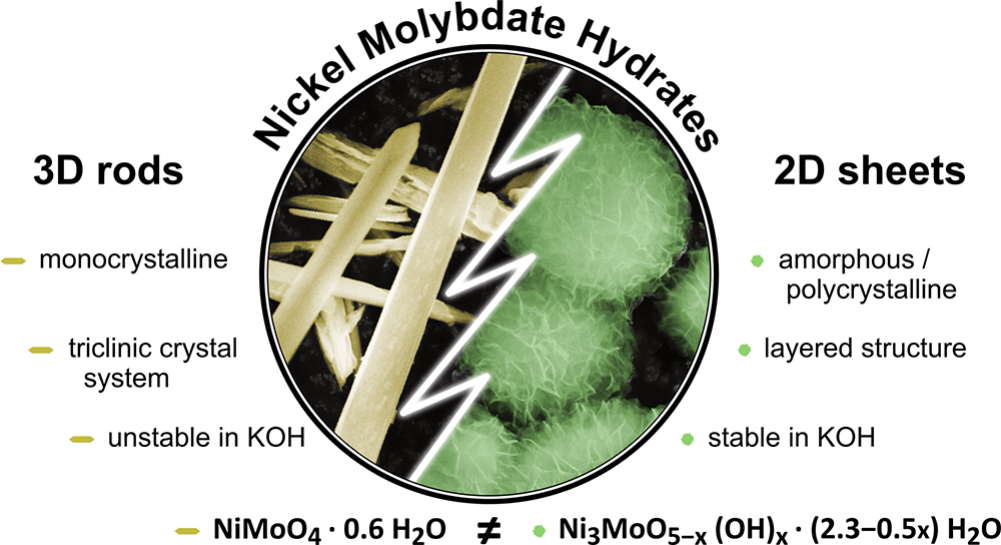

When electrocatalysts are prepared, modification of the morphology is a common strategy to enhance their electrocatalytic performance. In this work, we have examined and characterized nanorods (3D) and nanosheets (2D) of nickel molybdate hydrates, which previously have been treated as the same material with just a variation in morphology. We thoroughly investigated the materials and report that they contain fundamentally different compounds with different crystal structures, chemical compositions, and chemical stabilities. The 3D nanorod structure exhibits the chemical formula NiMoO_4_·0.6H_2_O and crystallizes in a triclinic system, whereas the 2D nanosheet structures can be rationalized with Ni_3_MoO_5–0.5*x*_(OH)_*x*_·(2.3 – 0.5*x*)H_2_O, with a mixed valence of both Ni and Mo, which enables a layered crystal structure. The difference in structure and composition is supported by X-ray photoelectron spectroscopy, ion beam analysis, thermogravimetric analysis, X-ray diffraction, electron diffraction, infrared spectroscopy, Raman spectroscopy, and magnetic measurements. The previously proposed crystal structure for the nickel molybdate hydrate nanorods from the literature needs to be reconsidered and is here refined by ab initio molecular dynamics on a quantum mechanical level using density functional theory calculations to reproduce the experimental findings. Because the material is frequently studied as an electrocatalyst or catalyst precursor and both structures can appear in the same synthesis, a clear distinction between the two compounds is necessary to assess the underlying structure-to-function relationship and targeted electrocatalytic properties.

## Abstract 2

This work characterizes and highlights the differences between nickel molybdate hydrates in nanorod and nanosheet structures, which are often used as precatalyst for highly active electrocatalysts but treated as the same material. Analysis of the elemental composition, crystal structure, magnetization, crystallinity, and chemical stability concludes that these materials are not morphological variations of one compound but two fundamentally different compounds. Distinguishing those compounds is expected to be pivotal for a correct attribution of their properties.

## Introduction

Global warming caused by additional greenhouse gases, such as carbon dioxide, introduced by our daily activities is an urgent concern that can be counteracted by a transition into a sustainable energy economy. In this context, alternative energy carriers are imperative that can enable renewable energy schemes and avoid net carbon dioxide emissions into our atmosphere.^1−4^ In recent years, hydrogen has been considered a promising energy carrier.^5^ Hydrogen is today mainly produced by natural gas reforming and water gas shift reaction, but rising effort is made to enhance the production of hydrogen using renewable electricity via electrocatalytic water splitting. In this process, electricity is utilized to split water into molecular hydrogen and oxygen.^6,7^ Using an electrocatalytic cell driven by renewable energy sources such as wind, hydro, or solar power, the produced hydrogen is carbon dioxide neutral, neglecting the emitted carbon dioxide evolved in the manufacturing of such devices. To drive this electrochemical process efficiently, catalysts for the hydrogen evolution reaction (HER) and oxygen evolution reaction (OER) are indispensable. Unfortunately, the most active and stable catalysts in an acidic environment contain platinum, iridium, ruthenium, and their oxides, which all are or comprise rare and expensive metals, which hamper the commercialization and widespread use of this technology.^8,9^ On the other hand, in alkaline media, cheap and abundant catalysts based on transition metals and their oxides, such as NiMo, NiFe layered double hydroxides (LDHs), NiO, NiMoP, NiCo, CoO-MoO_2_, CoFeO, Co-TiO_2_, CuTi, and Al_2_Mn_2.5_O_4_, exhibit high activities toward HER or OER.^5,8,10−16^ Some of the most efficient catalysts in alkaline media for HER are bimetallic nickel molybdenum compounds (NiMo),^10,16−23^ whereas one of the most efficient catalysts for OER in alkaline media is NiFe-LDH.^10,24−27^ Both catalysts can be synthesized via a nickel molybdate hydrate precursor,^17,21,28,29^ making this precursor structure highly relevant. Choi and co-workers have reported a highly active NiFe_2_O_4–*x*_/NiMoO_4_ nanowire oxygen evolution catalyst originated from a nickel molybdate hydrate nanorod structure with a remarkably low overpotential of 326 mV for a current density of 600 mA cm^–2^.^29^ As we will show later, the nickel molybdate hydrate nanosheet structure was also present in their work, whose signal was detected but not assigned. Zhang and co-workers reported a promising MoNi_4_ electrocatalyst for the hydrogen evolution reaction, which also originated from a nickel molybdate hydrate nanorod precursor.^17^ With an overpotential of 44 mV for a current density of 200 mA cm^–2^, this catalyst has shown very promising performance. However, from the scanning electron microscopy (SEM) images at an early synthesis stage, it seems also here that there could be sheet structures directly on the nickel foam substrate, which agrees with our previous observation.^30^

A challenge in more complex catalyst materials is the precise control of the as-prepared catalyst and resulting active catalyst as exemplified above. Various nanostructures of metal oxides are known to be easily accessible by hydrothermal syntheses, making this versatile approach in contrast to other synthesis methods highly interesting for developing different morphologies.^31−34^ However, the controlling factors for those shapes are often not clear. In our recent publication, we have suggested the strong influence of ramping temperature in preparing the nanostructure of nickel molybdate hydrates, obtaining different ratios of nanorods and nanosheets.^30^ Wang and co-workers proposed that the holding temperature during the hydrothermal synthesis would be an influencing factor on the nanostructure, observing nanorods for temperatures below 150 °C and nanosheets for a temperature of 180 °C.^35^ Cai and co-workers instead observed nanorods and nanospheres with nanosheet structures depending on the detailed synthesis mixture. While keeping the precursor concentrations, molybdenum source, and solvothermal time, and temperature constant, they obtained nanorods when using nickel nitrate hexahydrate precursor in an ethanol/water mixture but instead nanospheres with a nanosheet like morphology when using nickel acetate tetrahydrate in water.^36^ In direct contrast to this, Peng and co-workers obtained nanorods when using water as the solvent for their synthesis and nanosheet structures for a mixture of water and ethanol.^37^ Some studies point out that a more alkaline environment during the synthesis favors the formation of nanosheets, as seen when adding urea or ammonium hydroxide to the synthesis solution.^28,38,39^

In our previous publication, we reported that not only do the seemingly similar 3D (rod) and 2D (sheets assembled to flowers) nanostructures of nickel molybdate hydrates exhibit different stabilities in 1 M KOH and elemental compositions, but their different structure can also be distinguished by Raman spectroscopy, Fourier transformed infrared spectroscopy (FTIR), and X-ray diffraction (XRD).^30^ Distinguishing the nanostructures, the building block compounds, and elucidating their fundamental differences are pivotal to establish a structure-to-property relationship for catalysts in water-splitting devices. Even more, since the synthesis of nanorods on nickel foam seem often to result in the presence of both structures as visible in *in situ* Raman spectroscopy with bands corresponding to the nanosheet structure in syntheses with nanorods.^29,30,40^ Despite their different Raman spectra, XRD pattern, chemical stability, and elemental composition, the two structures occurring in the 2D and 3D morphologies have so far been accounted for as the same material and just characterized as nickel molybdate hydrate with different morphologies, without any clear distinction. Here we perform a thorough analysis of the two different structures by SEM, energy-dispersive X-ray spectroscopy (EDX), X-ray photoelectron spectroscopy (XPS), Raman spectroscopy, Fourier-transform infrared spectroscopy (FTIR), powder X-ray diffraction (PXRD), transmission electron microscopy (TEM) with selected area electron diffraction (SAED) and continuous rotation electron diffraction (cRED), thermogravimetric analysis (TGA), ion beam analysis (IBA) with Rutherford backscattering spectrometry (RBS) and time-of-flight elastic recoil detection analysis (ToF-ERDA), zero-field cooled and field cooled magnetic measurements (ZFC-FC), as well as isothermal magnetization curves, showing that those nanostructures correspond to fundamentally different compounds instead of being polymorphs of one nickel molybdate hydrate. Based on density-functional theory (DFT) calculations, we furthermore propose a more precise crystal structure of the nickel molybdate hydrate nanorod compound, whose simulated Raman spectrum is identical to the experimental result. It is expected that some of the detected fundamental differences of these precatalysts, such as the elemental composition or the morphology, will translate into the final catalyst and hence will have an effect on catalytic performance. Therefore, a scrutinous investigation of the two precatalyst compounds is of utmost importance for future investigations and development of the active catalysts, which goes beyond the scope of this work. With our thorough characterization, we hope that this work will act as a clarifying reference and inspiration for further studies in neighboring material systems.

## Experimental Section

### Materials

All materials are used as received without further purification. Nickel(II) nitrate hexahydrate (purum p.a., crystallized, ≥97.0% (KT)), sodium molybdate dihydrate (ACS reagent, ≥99%), and urea (puriss, ACS reagent ≥99.5%) were purchased from Sigma-Aldrich. Ammonium molybdate (para) tetrahydrate (99%) was provided by Alfa Aesar.

### Synthesis

Nickel molybdate hydrate nanorods (NMO-H_2_O-rods) were synthesized in a hydrothermal synthesis. In a representative synthesis, 0.01 M (NH_4_)_6_Mo_7_O_24_·4H_2_O (AHM) and 0.07 M Ni(NO_3_)_2_·6H_2_O were dissolved in 60 mL deionized water (DI). These concentrations were chosen to provide a 1:1 molar ratio between molybdenum and nickel, as for the 2D nanosheet synthesis below. The solution was transferred into a 100 mL Teflon lined stainless steel autoclave, heated up in a muffle furnace to 150 °C with 2 °C min^–1^, and held at this temperature for 6 h. After cooling to room temperature, the yellow precipitate was washed with DI three times followed by one time with ethanol, collected each time by centrifugation, and finally dried at 60 °C. It should be noted that the reaction solution after the synthesis was still clear green. To remove lattice water, the dry precipitate was further held at 200 °C for 8 h.

For nickel molybdate hydrate nanosheets (NMO-H_2_O-sheets), the synthesis route of Chen et al. was mostly followed.^28^ Herein 2 mmol Na_2_MoO_4_·2H_2_O and 2 mmol Ni(NO_3_)_2_·6H_2_O were dissolved in 60 mL DI. Then 8 mmol urea was added. After stirring for 30 min, the solution was transferred into a 100 mL Teflon lined stainless steel autoclave, heated up in a muffle furnace to 160 °C, and held for 8 h. Because no heating ramp was reported in the original paper, a rate of 10 °C min^–1^ was applied. After synthesis, the solution was clear and colorless with bright green precipitates. Those precipitates were also washed, collected, and dried as the NMO-H_2_O-rods above.

No uncommon hazards were noted.

### Characterization

A more detailed description is given in the Supporting Information. In short, the nanostructures were analyzed by scanning electron microscopy (SEM) with a ZEISS 1530 equipped with a Schottky field emission gun and an Oxford Instruments X-MaxN detector for energy-dispersive X-ray Spectroscopy (EDX). X-ray photoelectron spectroscopy (XPS) was performed with a Physical Electronics PHI Quantera II Scanning XPS Microprobe using monochromatic Al Kα X-rays with 1486.6 eV. Powder X-ray diffraction (PXRD) was done using a Bruker D8 Advance diffractometer with a Cu Kα radiation. Transmission electron microscopy (TEM) was conducted with a JEOL JEM-2100F and a ThermoFisher Themis Z with a Schottky-type field emission gun. Zero field cooled and field cooled (ZFC-FC) and isothermal field-dependent magnetization loops were carried out using an MPMS XL SQUID magnetometer. Rutherford backscattering spectrometry (RBS) and time-of-flight elastic recoil detection analysis (ToF-ERDA) were conducted in the Tandem Laboratory of Uppsala University using a 2 MeV ^4^He^+^ and a 44 MeV ^127^I^10+^ beam, respectively, bombarding a compressed pellet of the nanostructures. Thermogravimetric analysis (TGA) was performed with a TA Instruments TGA Q500 instrument. Raman spectroscopy was conducted with a Renishaw Reflex (Invia) Raman spectrometer and a Renishaw Qontor (Invia) Raman spectrometer using a frequency doubled Nd:YAG 532 nm laser and a Renishaw HPNIR 785 semiconductor laser source, respectively. Attenuated total reflection Fourier-transform infrared spectroscopy (ATR-FTIR) was carried out with a Bruker Vertex 70v Spectrometer equipped with a A225/Q Platinum ATR diamond unit. Density-functional theory (DFT) calculations were performed using the projected augmented wave (PAW) method to solve the Kohn–Shan equations as implemented in the Vienna Ab initio Simulation Package (VASP).^41,42^ The spin-polarized generalized gradient approximation has been used with the Perdew, Burke, and Ernzerhof (PBE) parametrization to describe the exchange and correlation term of the Kohn–Sham Hamiltonian.^43^ Plane waves were expanded to an energy cutoff of 600 eV, whereas Brillouin sampling was performed in a reciprocal grid of 4 × 4 × 2 for the density of states calculations and 2 × 2 × 1 for the structural relaxation. To include the strong correlation of the 3d electrons of the Ni, the approach implemented by Dudarev et al. was employed.^44^ In this level of theory, an effective *U* value, i.e., *U*_eff_= *U* – *J*, was considered instead of separately considering the Hubbard repulsion term *U* and the exchange term *J*. The *U*_eff_ parameter of the 3d Ni states was taken as 6.0 eV from refs (45−47). Ferromagnetic ordering (FM) has been employed for all calculations carried out here. Moreover, the DFT + D3 approach was used to take into account van der Waals interaction.^48,49^ Force convergence was set to 0.01 eV/Å, whereas energy convergence was set to1 0^–4^ eV. Water positions inside the crystal lattice of NiMoO_4_ were optimized using a homemade minima hopping (MH) global optimization algorithm.^50^ There, temperature was employed to overcome energy barriers and explore the energy landscape. Short molecular dynamics (MD) simulations were used to escape local minima followed by local optimizations using on-the-fly adjusted parameters. The *ab initio* MD (AIMD) parts of the MH were performed in the microcanonical ensemble (NVE) with a velocity Verlet algorithm and a time step of 0.5 fs. The MH algorithm started at 600 K with an initial energy threshold of 0.5 eV. The Raman intensities were calculated for a 2 × 2 × 1 supercell containing 16 formula units of NiMoO_4_(H_2_O)_0.75_. We would here like to emphasize that because the forces and thus Raman response depend on the Hessian, which is the second derivative of the position of the nuclei, more strict convergence criteria and more computationally demanding calculations are necessary in comparison to electronic structure calculations, which typically converge before the detailed positions of the nuclei. Second derivatives of the total energy with respect to the ion’s displacement were calculated using the finite difference approach to accessing the phonon modes and frequencies at the gamma point. For this part, the structure was reoptimized for a force convergence of 0.001 eV/Å together with an energy convergence of 10^–8^ eV. Finite differences in the direction of each vibrational mode were used to estimate the difference in the macroscopic dielectric tensor and, hence, access their relative Raman intensities.^51^

## Results and Discussion

Because, in total, two different materials and several characterization techniques for each material have been used, which potentially could cause confusion, we refer the mindful reader in times of need for clarification to Chart S1, where a schematic overview of the materials and techniques is given, including the main finding of each technique.

**Figure 1:**
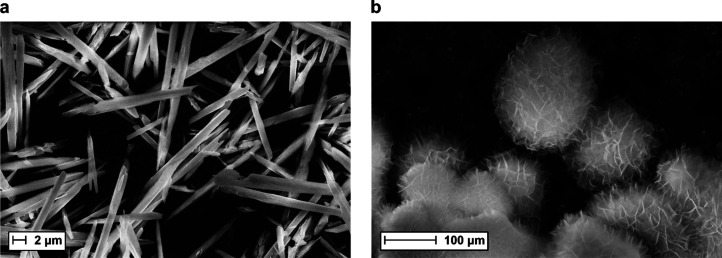
Secondary electron (SE) images of the different as-synthesized nanostructures suggest the successful exclusive synthesis of both nanostructures. (a) Nanorods for NMO-H_2_O-rods. (b) Nanosheets of NMO-H_2_O-sheets assembled into a flower microstructure.

The two nickel molybdate hydrate (NMO-H_2_O) nanostructures were synthesized as powders in a hydrothermal synthesis, as described in detail in the SI. Their preferential nanostructure shapes were confirmed by scanning electron microscopy (SEM) in Figure 1 (and Figures S1 and S2). Secondary electron imaging of the nickel molybdate hydrate rods (NMO-H_2_O-rods) showed rods with a diameter of around 1–2 μm and several μm in length. Small particles observed in the images were attributed to fractures of rods and not to another nanostructure. The nickel molybdate hydrate sheet (NMO-H_2_O-sheets) nanostructure exhibited 2D thin nanosheet shapes, whose thickness was estimated by transmission electron microscopy (TEM) below. The nanosheets assembled in a random orientation to microscopic spherical flower-like superstructures, which themselves arranged to form larger clusters.

### Elemental Analysis

Energy-dispersive X-ray spectroscopy (EDX) was utilized to probe the distribution of elements in the different nanostructures. In both the structures, Ni, Mo, and O were homogeneously dispersed within the level of resolution of the electron microscope (Figures S3 and S4). The atomic concentration of nickel-to-molybdenum for the 2D structures, however, increased to approximately 3:1 compared to an equimolar ratio for the nanorods extending in 3D. This was further corroborated with elemental analysis by X-ray photoelectron spectroscopy (XPS) and ion beam analysis (IBA) techniques below.

XPS confirmed the presence of only Ni, Mo, O, and C in both nanostructures, however with a significantly different element concentration between the nanostructures as already indicated by EDX (Figures S5 and S6). High-resolution XPS of Ni 2p, Mo 3d, and O 1s suggested 13.4 at. % nickel, 14.9 at. % molybdenum, and 71.7 at. % oxygen for 3D NMO-H_2_O-rods and 24.3 at. % nickel, 7.7 at. % molybdenum, and 68.0 at. % oxygen for 2D NMO-H_2_O-sheets, proving a clear elemental difference between those two nanostructures (Table S1). However, because the carbon concentration was too high to be only from adventitious carbon, along with the detection of Si from the Leit tab, the contribution of the Leit tab to the detected oxygen concentration cannot be neglected. Atomic concentration excluding oxygen from the Leit tab is presented in the last column of Table S1. All binding energies were corrected versus adventitious carbon C 1s at 284.8 eV.

**Figure 2:**
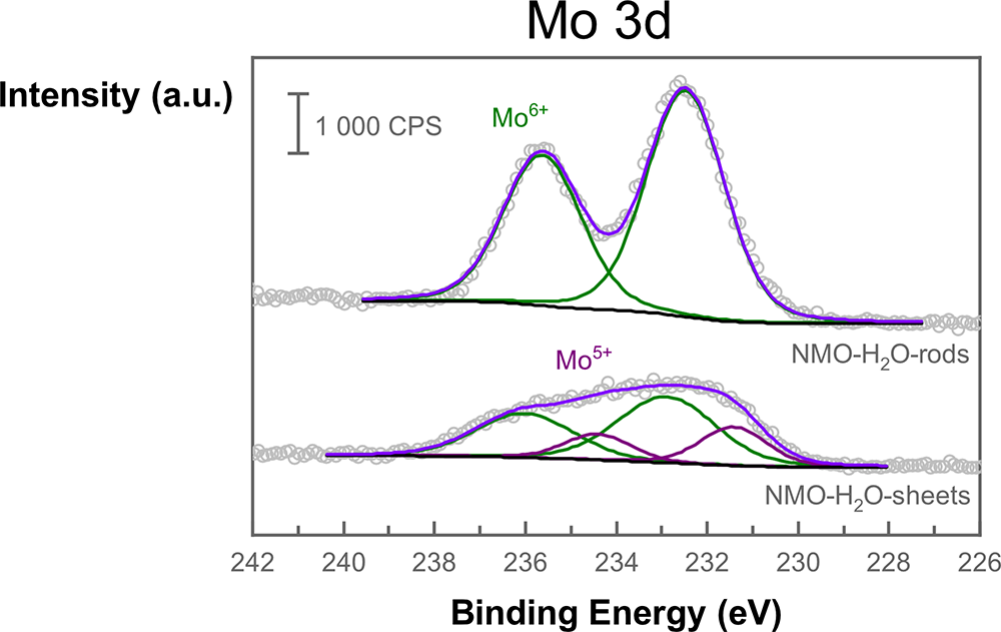
Molybdenum 3D high-resolution spectra for NMO-H_2_O-rods above and NMO-H_2_O-sheets below indicate the presence of an additional molybdenum oxidation state present in NMO-H_2_O-sheets. The green fit corresponds to Mo^6+^, whereas the purple fit represents Mo^5+^. The violet fit corresponds to the envelope.

High-resolution XPS of Ni 2p for 3D NMO-H_2_O-rods could be separated into two contributions for Ni 2p_3/2_ with binding energies at 856.1 and 858.2 eV and the corresponding peaks at Ni 2p_1/2_ at 873.7 and 876.1 eV (Figure S5b). The two contributions could originate from different Ni environments as shown below in the optimized crystal structure. However, it should also be noted that curve fitting the Ni 2p orbital is rather complex and sometimes requires several contributions,^52,53^ which could explain the slightly too large detected spin–orbital splitting of 17.6 and 17.9 eV, respectively. Satellites of the Ni 2p spectrum were detected at 862.3 and 880.2 eV. The Mo 3d spectrum of the rods can be curve-fitted to Mo 3d_5/2_ at 232.5 eV and Mo 3d_3/2_ at 235.6 eV, representing Mo^6+^ and agreeing with previously reported binding energies (Figure S5c).^54,55^ The O 1s spectra show metal-oxide contribution at 530.7 eV, which represents an envelope of Ni–O and Mo–O, as well as O–H contribution at 532.9 eV, which probably includes some O–C contribution (Figure S5d). The high-resolution Ni 2p spectra for 2D NMO-H_2_O-sheets could be well fitted with a contribution at 856.0 and 873.7 eV for Ni 2p_3/2_ and Ni 2p_1/2_, respectively (Figure S6b). Because the oxidation state and the coordination of Ni in this compound are unclear as further explained below, a faithful curve fitting of the Ni 2p spectrum is not possible. Additional satellite peaks were detected at 861.7 and 879.9 eV. The Mo 3d spectrum of NMO-H_2_O-sheets showed a significant difference compared to the spectrum of the rods. The first doublet at 232.9 and 236.0 eV for Mo 3d_5/2_ and 3d_3/2_, respectively, can be contributed to Mo^6+^, whereas the second doublet at 231.4 and 234.4 eV for Mo 3d_5/2_ and 3d_3/2_, respectively, might originate from Mo^5+^, indicating a mixed valence molybdenum in this nanostructure (Figure 2 and Figure S6c).^55^ As for the rod structure, also NMO-H_2_O-sheets showed an O 1s spectrum with O–H and metal-oxide contributions at 532.1 and 530.6 eV, respectively (Figure S6d). The exact binding energies are difficult to determine because the magnitudes of contributions from O–C, O vacancies, Ni–O, and Mo–O are uncertain. The shift to slightly lower binding energies could be attributed to a larger Ni–O contribution, which is reported at lower binding energies compared to Mo–O.^54,56^

**Figure 3:**
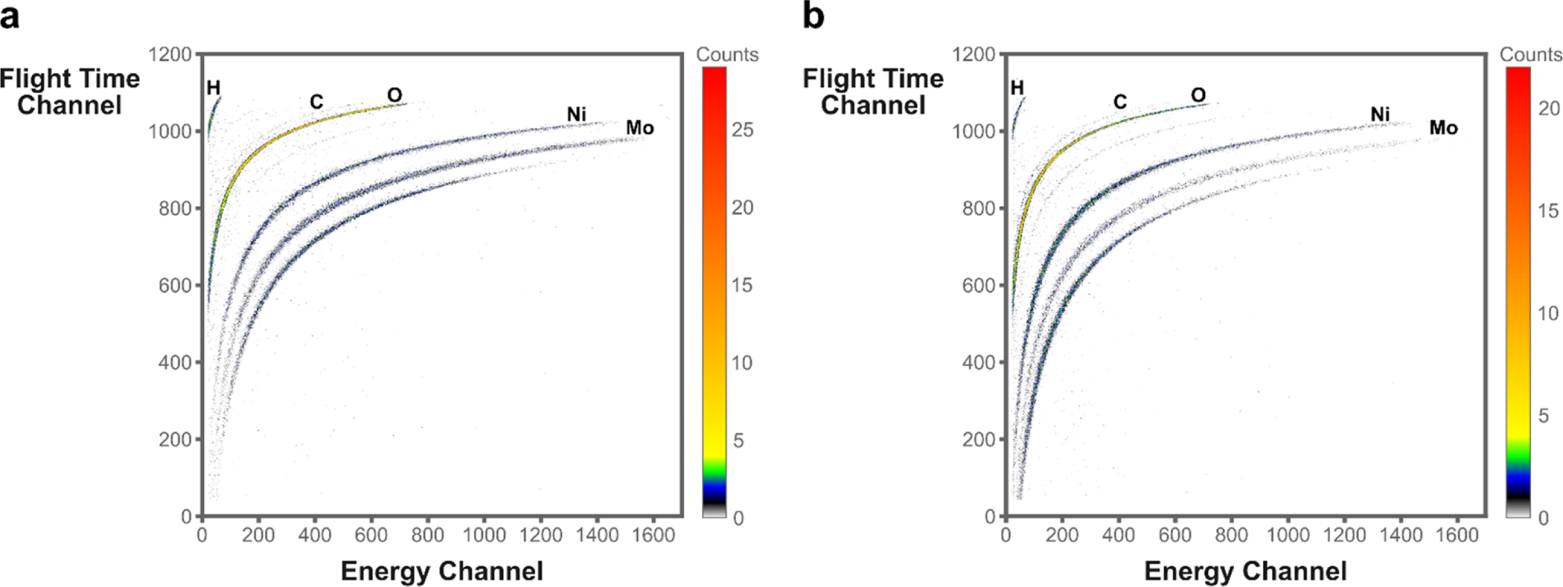
ToF-ERDA analysis of NMO-H_2_O-rods (a) and NMO-H_2_O-sheets (b) shows different elemental compositions for the two nanostructures, further quantified by depth profiling. The unlabeled traces to the lowest right are from iodine used for the bombardment of the samples.

IBA techniques were used to further determine the atomic composition of both nanostructures. Time-of-flight elastic recoil detection analysis (ToF-ERDA) (Figure 3) was utilized to probe the hydrogen, oxygen, nickel, and molybdenum atomic concentration by depth profiling (Figure S7). A significant ion-beam-induced hydrogen release was detected for the 2D NMO-H_2_O-sheets, and the calculated composition was compensated accordingly (Figure S8). Interestingly, no ion-beam-induced hydrogen release was detected for 3D NMO-H_2_O-rods. The atomic concentration was further refined by adjusting the nickel-to-molybdenum ratio according to the results of the Rutherford backscattering spectrometry (RBS) (Figure S9). The calculated atomic composition for 3D NMO-H_2_O-rods was detected as 13.44 ± 0.43 at. % nickel, 13.56 ± 0.45 at. % molybdenum, 64.58 ± 0.91 at. % oxygen, and 8.42 ± 0.50 at. % hydrogen. For 2D NMO-H_2_O- sheets, 22.29 ± 0.29 at. % nickel, 7.36 ± 0.72 at. % molybdenum, 53.81 ± 0.20 at. % oxygen, and 16.54 ± 0.92 at. % hydrogen were calculated. It should be noted that it is not possible to exactly determine the concentration of hydrogen with those techniques but should be taken as verification of the presence and as an indication of the difference in magnitude between the samples. The difference in composition compared to the XPS analysis is due to the surface sensitivity of the latter technique. As visible in Figure S7, the atomic concentration at low depths does not fully represent the concentration in the bulk, hence leading to slightly different quantification on the surface.

Thermogravimetric analyses (TGAs) were conducted on the as-synthesized powders before and after drying at 200 °C (Figure S10). The overall loss of mass during heating for NMO-H_2_O-sheets (−16.77 wt %) was significantly higher than that for NMO-H_2_O-rods (−6.45 wt %). Assuming that the loss in this temperature range is only assigned to the removal of water, this indicates a higher amount of water in the nanosheet structure compared to the rods, as later quantified more precisely. For the NMO-H_2_O-rods, different temperature regions could be identified for the loss of reversibly bonded water (100–200 °C) and removal of crystal water (CW) during the phase transformation (200–300 °C). The small additional loss of mass at around 350 °C could be due to the loss of reversibly bonded water of the transformed phase, as proposed by Rodriguez et al.^57^ The difference in temperature at which these processes were detected—compared to the mentioned work—can be attributed to the slower heating ramp in the TGA.

For NMO-H_2_O-sheets, a rapid loss of mass was observed before 100 °C, attributed to reversibly bonded water. Between approximately 210 and 400 °C, the majority of loss of mass was observed, attributed to the removal of CW or dehydration of hydroxides, which likely is present in a layered structure as proposed below. The temperature derivative in Figure S10b,d seemed to be a convolution of two or three regions, agreeing that the loss might happen in different processes in this temperature range. As described in the SI, during the drying step at 200 °C for 8 h, no significant amount of CW or hydroxides was removed, with was further corroborated by the unchanged Raman spectra before and after the drying step (Figure S17).

Combining the elemental concentration of nickel, molybdenum, and oxygen from IBA and the amount of crystal water from TGA led to a proposed stoichiometry of Ni_1.0_Mo_1.0_O_4.0_·0.6 H_2_O for the 3D NMO-H_2_O-rods and Ni_3.0_Mo_1.0_O_4.1–0.5*x*_(OH)_*x*_·(2.2 – 0.5*x*)H_2_O for 2D NMO-H_2_O-sheets, assuming that IBA results represent the material observed in TGA at 20 °C with reversibly bonded water (for a more detailed description of the calculation, see Note S1). However, the extent of reversibly bonded water present during the ion beam analysis is unclear. Hence, the stochiometric formulas were recalculated assuming that IBA represents only NMO-H_2_O without reversibly bonded water. In this case, Ni_1.0_Mo_1.0_O_4.2_·0.6H_2_O and Ni_3.0_Mo_1.0_O_5.0–0.5*x*_(OH)_*x*_·(2.3 – *x*)H_2_O were obtained for NMO-H_2_O-rods and NMO-H_2_O-sheets, respectively. The actual stoichiometries may lie in between the proposed values.

To fulfill charge neutrality, the oxidation states for Ni and Mo in 3D NMO-H_2_O-rods should be 2+ and 6+, respectively. Molybdenum in 6+ was confirmed by high-resolution XPS. For 2D NMO-H_2_O-sheets, however, molybdenum was detected to have contributions of Mo^5+^ and Mo^6+^ in high-resolution XPS of Mo 3d (Figure 2 and Figure S6c), meaning that nickel would also have to be present in a mixed valence oxidation state. More precisely, it would have to lay between Ni^0^ and Ni^1+^ to reach charge neutrality for Ni_3.0_Mo_1.0_O_4.1–0.5*x*_(OH)_*x*_·(2.2 – *x*)H_2_O and between Ni^1+^ and Ni^2+^ for Ni_3.0_Mo_1.0_O_5.0–0.5*x*_(OH)_*x*_·(2.3 – *x*)H_2_O. Because the oxidation state of nickel in layered hydroxides is usually closer to 2+ and a nickel oxidation state of around 2+ was further proposed by magnetic measurements below, we believe that this chemical formula for the NMO-H_2_O-sheets seems therefore more reasonable. In addition, TGA proposed on average a 1.92 times higher hydrogen concentration in NMO-H_2_O-sheets compared to NMO-H_2_O-rods, which is very close to the 1.96 times higher hydrogen concentration proposed by IBA.

**Figure 4:**
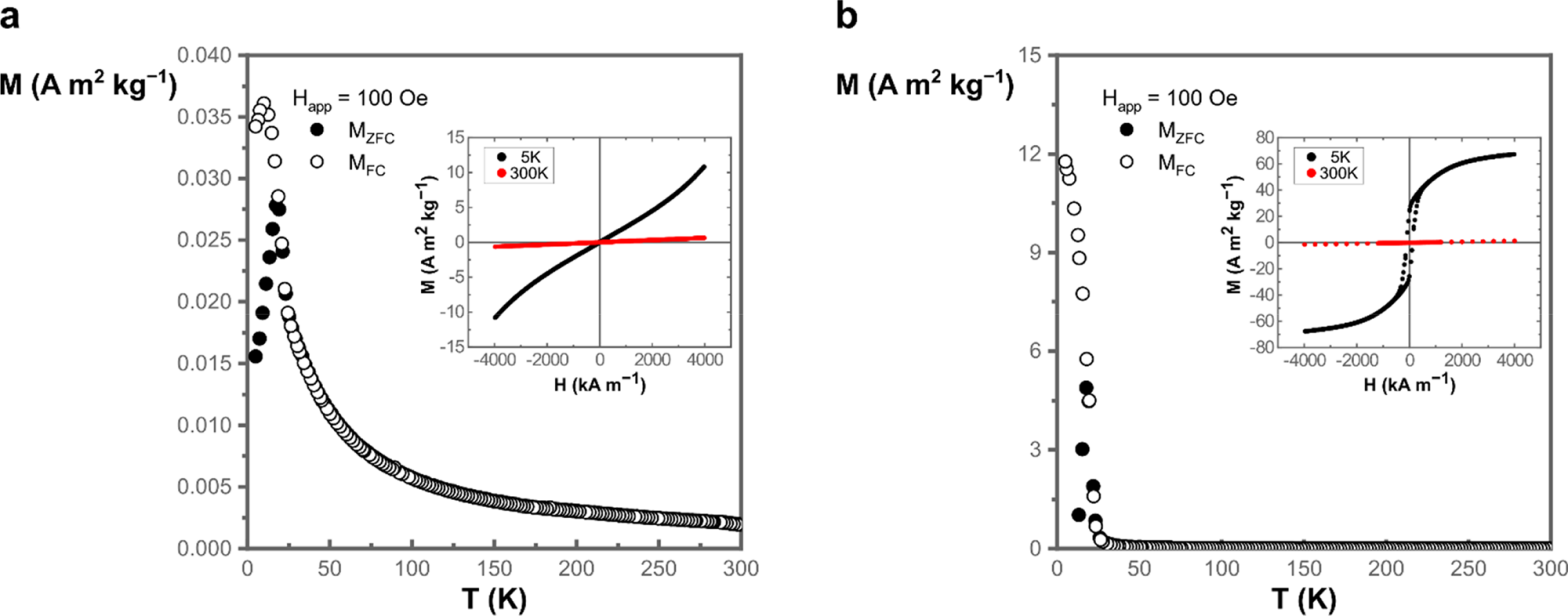
Zero field cooled–field cooled (ZFC-FC) measurements between 5 and 300 K for (a) NMO-H_2_O-rods and (b) NMO-H_2_O-sheets. For FC, a magnetic field with a strength of 100 Oe was applied. In the insets are the isothermal magnetization loops at 5 and 300 K for the corresponding compound. The different responses in magnetization and magnetic behavior corroborate the fundamental difference between those nanostructures.

With zero field cooled (ZFC) and field cooled (FC) measurements, the temperature dependence of the magnetization was investigated. In the ZFC magnetization, a transition from paramagnetic to antiferromagnetic behavior was observed for NMO-H_2_O-rods below 17 K, illustrated by the characteristic cusp at that temperature (Figure 4a), akin to that observed in similar systems.^58−60^ In the presence of a small magnetic field in FC, this cusp was shifted slightly to a lower temperature, and it suggests an excess moment superimposed to the antiferromagnetic state.^61^ The assignment of the paramagnetic and antiferromagnetic regime was strengthened by the isothermal field-dependent magnetization loops at 300 and 5 K in the inset in Figure 4a and with the Curie–Weiss temperature θ < 0 K (−5 K) (Figure S11).

Interestingly, the magnetization at 5 K revealed a change of slope for an applied magnetic field above approximately 3000 kA m^–1^, which may reflect the transition into a metamagnetic phase.^62^ More investigations are required to fully confirm this observation, but it is out of the scope of this study. Derived from the ZFC magnetization data, the effective magnetic moment μ_eff_ of nickel could be estimated from the obtained Curie constant as μ_eff_ = 3.3 μ_B_, with μ_B_ as the Bohr magneton (Figure S11). This value lies close to the paramagnetic moment of Ni^2+^ in an octahedral ligand field in the 3D rods,^63^ which would agree with the required oxidation state for charge neutrality of the compound as well as partially with the proposed coordination of Ni in the structure.^64^

For the 2D NMO-H_2_O-sheets, a cusp is observed in ZFC in the same low-temperature region as for the rods (Figure 4b) with a significantly higher magnetization. The sharp transition in the *M* vs *T* curve suggests a magnetic ordering below 25 K. The isothermal field-dependent magnetization curves at 300 and 5 K in the inset agree with this assignment. At 5 K, the curve reveals open loops near the origin with a coercive field of *H*_c_ = 120 kA m^–1^ and an absence of magnetic saturation. The data above 40 K follow a Curie–Weiss behavior with a (ferromagnetic) positive θ ∼+34 K. The obtained effective moment in this case is about 2.6 μ_B_ (Figure S13e), which is lower than for the previous compound and instead close to the spin-only value of μ_eff_ for Ni^2+^ (*S* = 1). However, because a mixed valence nickel is required for charge neutralization and the coordination of Ni in the 2D NMO-H_2_O-sheets is unclear, a faithful assignment of the oxidation state of nickel in the 2D sheets is not possible from the data.

### Structural Characterization

**Figure 5:**
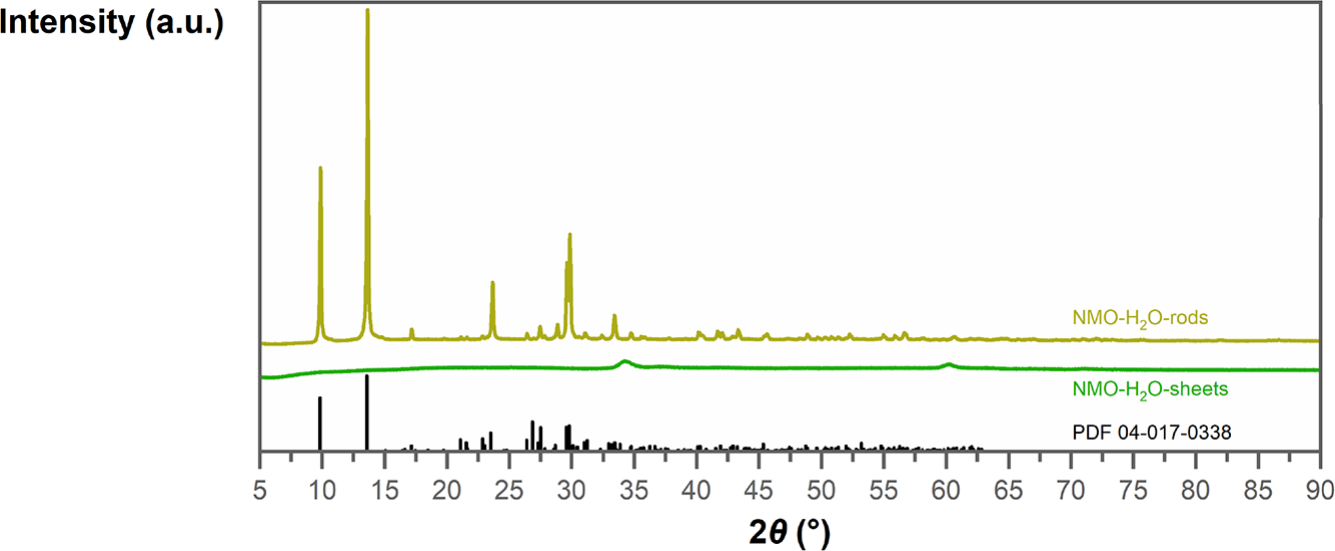
Powder X-ray diffractograms of the as-synthesized NMO-H_2_O nanostructures. 3D NMO-H_2_O-rods in yellow show clear reflexes, and 2D NMO-H_2_O-sheets in green show broad and low-intensity reflexes, reminding of a poorly crystalline material. The reference diffractogram of nickel molybdate hydrate (PDF 04-017-0338) in black bars shows a good fit with NMO-H_2_O-rods.

Powder X-ray diffraction (PXRD) patterns of the as-synthesized materials, shown in Figure 5, exhibited a fundamental difference between the two nanostructures. The 3D NMO-H_2_O-rods seem highly crystalline with clear reflections. The main peaks at 9.9, 13.6, 23.7, 29.6, and 29.8° fit to nickel molybdate hydrate described in PDF 04-017-0338 by Eda et al.^64^ Fitting the obtained PXRD pattern by the Le Bail method to the triclinic crystal structure of Eda et al. resulted in a unit cell with *a* = 6.7412(2) Å, *b* = 6.8964(2) Å, *c* = 9.2406(2) Å, α = 76.371(3) °, β = 84.278(2) °, and γ = 74.437(4) °, which are very close to the proposed unit cell parameters. However, both the PXRD and the electron diffraction patterns below illustrated some discrepancies with the proposed crystal structure. The 2D NMO-H_2_O-sheets exhibit a diffractogram with broad peaks at 34.3 and 60.2°, which agree with diffraction peaks for this nanostructure detected in previous publications.^30,65^

Xie and co-workers proposed this nanostructure being α-Ni(OH)_2_ (JCPDS No. 38-0715) intercalated by MoO_4_^2–^ anions or water molecules,^65^ agreeing with Han et al., who proposed a nickel–iron LDH intercalated with molybdate anions for their material.^39^ In addition, Gunjakar et al. reported the synthesis of nickel hydroxide sheets intercalated by polyoxovanadate anions with a very similar PXRD pattern.^66^ However, neither Raman spectroscopy nor Fourier-transformed infrared spectroscopy (FTIR) could detect α-Ni(OH)_2_ vibrations.^67−69^ Nevertheless, in accordance with the previous literature, it seems highly likely that the obtained diffractogram could be rationalized with layered Ni(OH)_2_ sheets with intercalated or incorporated molybdate ions. The absence of strong nanorod X-ray diffractions indicates the exclusive synthesis of the 2D nanosheet structure. With Raman spectra on the same samples before and after PXRD analysis, no phase transformation during the PXRD measurement was verified (Figure S18).

**Figure 6:**
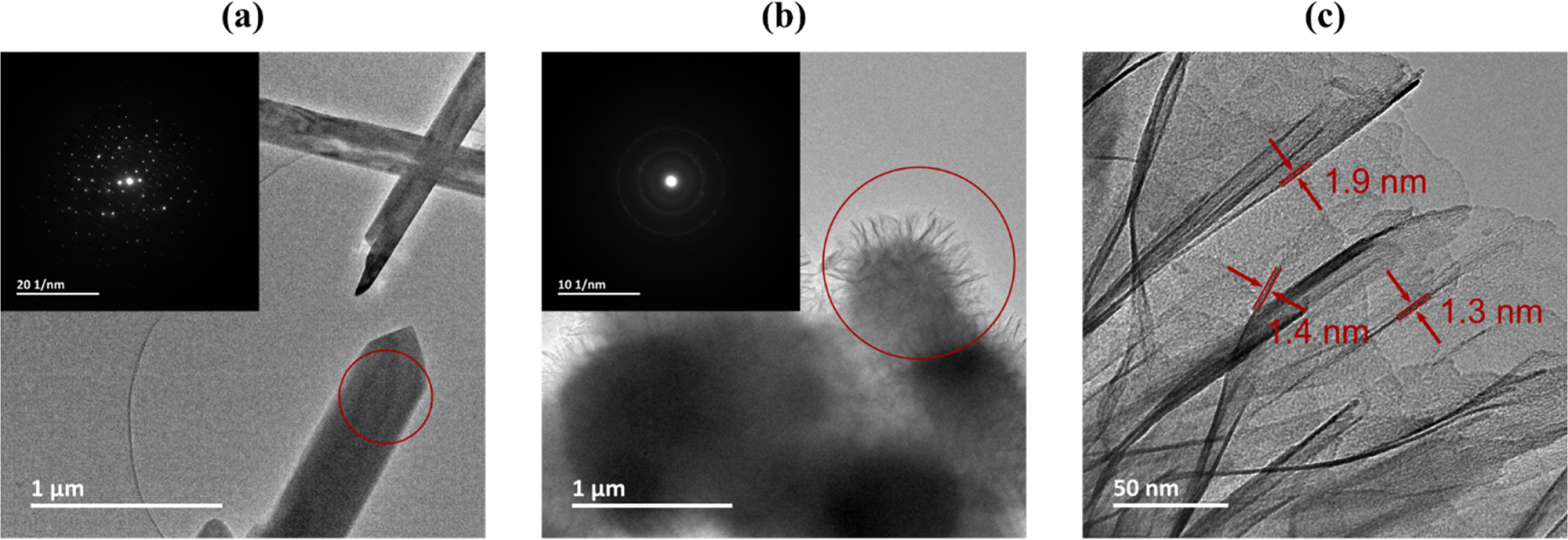
TEM analysis of NMO-H_2_O nanostructures. (a) TEM image of rods with the red circle indicating where the SAED in the inset was taken from, showing well-defined diffraction spots. (b) TEM image and SAED in the inset of the sheets. The red circle shows the area of SAED acquisition, and the SAED shows sharp diffraction rings. The SAED patterns’ brightness and contrast were adjusted to enhance the visibility of the diffraction spots and rings. (c) TEM image of the sheets with an upper estimation of sheet thickness.

With transmission electron microscopy (TEM) images, the exclusive synthesis of 3D rod structures for NMO-H_2_O-rods was corroborated (Figure S14). At high resolution, not only lattice fringes in the bulk were detected but also a different orientation of those fringes on the surface of the rods (Figure S14c,d). Saito and co-workers proposed this change to originate from the incline of the 3D rod structure.^70^ However, in addition to the suggested incline, surface damages on the rods were already observed for some rods in SEM, and hence, it is uncertain if those fringes are due to the incline, defects, possible localized surface phases, or a mixture of those. With selective area electron diffraction (SAED) on NMO-H_2_O-rods, only distinct diffraction spots were observed, proposing the presence of a single crystal, which would counterargue a second phase on the surface (Figure 6a). However, the reflections could not be attributed to any plane of the reported crystal structure for NiMoO_4_·3/4H_2_O described in PDF 04-017-0338, strengthening the suggestion of a discrepancy to the reported crystal structure for this material. Reconstructed 3D-electron diffraction in reciprocal space obtained by continuous rotation electron diffraction (cRED) was utilized for further insight (see Video S1). The refinement crystal structure suggested a triclinic system with space group *P*–1 and unit cell parameters given in Figure S15. It is worth noting that the crystal structure proposed by cRED was derived by assuming oxygen coordinating nickel octahedrally and molybdenum tetrahedrally (Figure S15). However, the obtained crystal structure was not well compatible with the acquired PXRD pattern. The advantages of cRED are to collect 3D electron diffraction of nanocrystals by TEM and to use the acquired reconstructed reciprocal space to determine the space group and structure, as well as structure solution and refinement. But it also has physical limitations, such as strong dynamical effect and decay of scattering intensity along reciprocal space; these might cause inaccuracy of the structure refinement. Nevertheless, both PXRD and electron diffraction results agreed with the proposed triclinic crystal structure with space group *P*–1 from PDF 04-017-0338.

For the 2D NMO-H_2_O-sheets, only small crystalline regions with different orientations were detected (Figure S16b–d). Their crystallinity was further confirmed by SAED with sharp diffraction rings (Figure 6b). The thickness of the sheets was estimated to be below 1.9 nm (Figure 6c), indicating a nearly ideal 2D structure. The thickness analysis is based on the measured thickness of a sheet rolling into the optical axis. Because it can be assumed that the sheet was not positioned perfectly in plane with the optical axis, the extracted thickness approximates an upper limit of the thickness.

### Vibrational Spectroscopy

**Figure 7:**
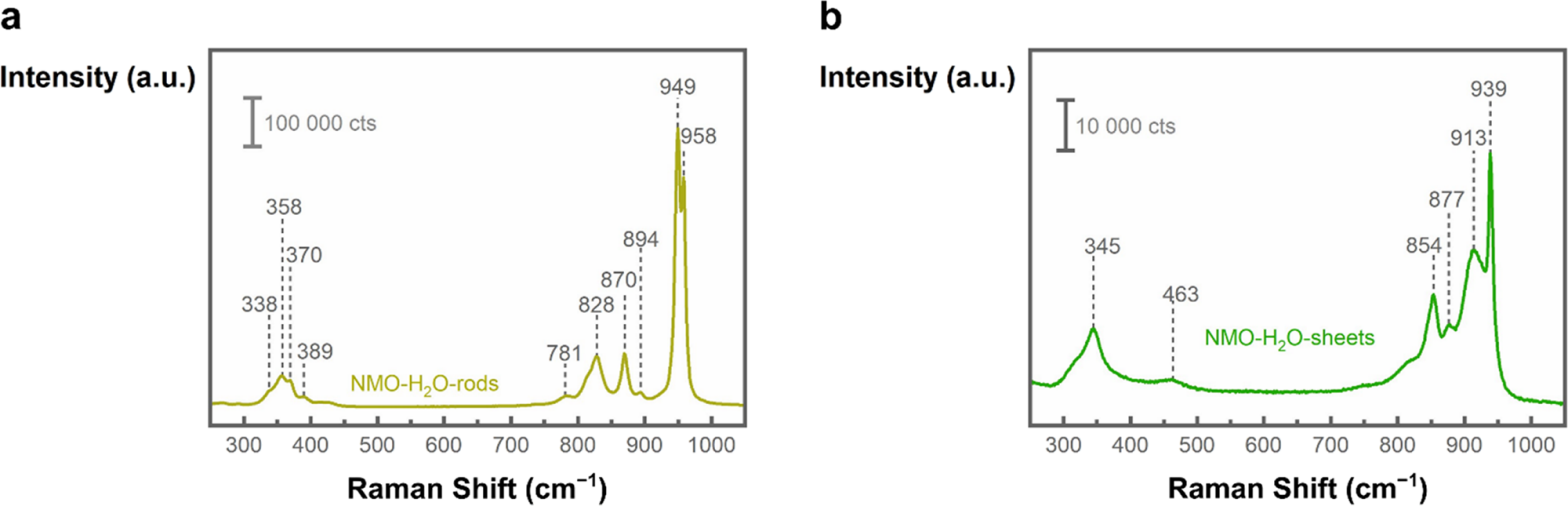
Raman spectra of the as-synthesized nanostructures acquired with 532 nm laser excitation. (a) NMO-H_2_O-rods. (b) NMO-H_2_O-sheets. The numbers represent the wavenumbers of the detected Raman bands and show different vibration features for NMO-H_2_O-rods and NMO-H_2_O sheets.

Raman spectroscopy in Figure 7a showed vibration modes for the NMO-H_2_O-rods with two strong peaks at 958 and 949 cm^–1^ as well as peaks at 870, 828, 370, and 358 cm^–1^ and weaker peaks at 894, 781, 389, and 338 cm^–1^. The peaks at 949, 894, 870, 828, 358, and 338 cm^–1^ are in the range of previously reported wavenumbers.^30,71^ In addition, the peaks at 958, 781, 389, and 370 cm^–1^ were previously observed as shoulder or minor peaks, but not assigned.^30^ The vibrations at 781, and 389 cm^–1^ might present the first overtone and the corresponding fundamental mode of the same vibration. It is known that overtones and differential tones can complicate the assignment of wavenumbers to vibration modes.^72^ It has been proposed that the vibrations between approximately 800 and 1000 cm^–1^ originate from symmetric and asymmetric Mo–O stretching modes, whereas the vibrations at lower wavenumbers can be attributed to bending modes.^73,74^ Fan et al. proposed in their work that the detected vibrations around 959 and 947 cm^–1^ originate from MoO_6_ octahedra of α-NiMoO_4_ and MoO_4_ tetrahedra of β-NiMoO_4_, respectively.^56^ Because β-NiMoO_4_ is reported to be unstable at room temperature^57^ and α-NiMoO_4_ should exhibit a peak corresponding to Ni–O–Mo stretching at around 700 cm^–1^, a mixed structure of α-NiMoO_4_ and β-NiMoO_4_ is hence less likely.

The 2D NMO-H_2_O-sheets in Figure 7b exhibited a strong band at 939 cm^–1^, a broad one at 913 cm^–1^, as well as peaks at 877, 854, 463, and 345 cm^–1^. The detected peaks are in agreement with previously reported values.^30,39^ The origin of the peak at 877 cm^–1^ is so far unclear but could be due to impurities or trace elements of the chemicals used in the synthesis or an overlay of different vibration modes.^75^ The Raman band at 463 cm^–1^ could come from a Ni(OH)_2_ vibration mode.^76^ However, the missing strong Raman bands for the Ni–O stretching modes at 504 or 534 cm^–1^ suggest the absence of α-Ni(OH)_2_.^67−69^ Variations in the peak intensity of the Raman band at 913 cm^–1^ compared to the one at 939 cm^–1^ can originate from different molybdenum environments. Possibly, the different Raman shifts are caused by Mo–O vibrations at the edges of the 2D sheets, within the plane of the sheets, from areas in which the sheets assembled to flowers or from grain boundaries or amorphous regions between the small crystalline grains. Different ratios of these environments might lead to different intensity ratios of the corresponding Raman bands.

It should be noted that the intensity of the Raman bands between the NMO-H_2_O-sheets and NMO-H_2_O-rods differed by roughly 1 order of magnitude (Figure S19), indicating why 2D NMO-H_2_O-sheet vibrations might be overseen in Raman spectra with nanorods present. To exclude the contribution of a possible resonance Raman effect, spectra were additionally acquired with a 785 nm laser excitation, which showed the same Raman bands, hence verifying the absence of resonance Raman effects for 532 nm excited Raman spectra (Figure S20). The additional bands detected at approximately 1300 cm^–1^ in the spectra with 785 nm excitation might originate from magnons or plasmons. It is unclear at this point why they were not detected in the 532 nm excitation spectra.

Both structures were immersed in 1 M KOH for selective molybdenum leaching. As reported earlier, molybdenum from the nanorod structure leaches out, causing the Mo–O vibrations of the nanorod to vanish.^30^ On the other hand, molybdenum remains in the 2D nanosheet structure as indicated by the unchanged Mo–O vibrations (Figure S21).^30^ The absence of nanosheet bands in the spectra of the nanorod after selective molybdenum leaching further supports the isolated synthesis of nanorods (Figure S22). Molybdenum leaching highlights the superior chemical stability of 2D NMO-H_2_O-sheets over rods in 1 M KOH and offers a strong route to verify the absence of NMO-H_2_O-sheets in a possibly mixed 2D and 3D NMO-H_2_O system.

Attenuated total reflection Fourier-transform infrared spectroscopy (ATR-FTIR) confirmed the presence of water with absorbance in the region of 3600– 3000 cm^–1^ and at around 1630 cm^–1^ as shown in Figure S23a. In the fingerprint region visible in Figure S23b, NMO-H_2_O-rods exhibit absorption bands at 963, 911, 882, 851, 818, 731, and 447 cm^–1^. NMO-H_2_O-sheets in comparison showed absorption peaks at 931, 868, 806, 685, and 486 cm^–1^. The wavenumbers at 963, 911, 882, 818, 731, and 447 cm^–1^ for the rods and 931, 868, 806, and 685 cm^–1^ for the sheets are in accordance with previously reported values.^30,77−79^ The vibration at 851 cm^–1^ for the rods was so far not possible to assign, whereas the vibration at 486 cm^–1^ of the sheets is considered to originate in a Ni–O mode.^80,81^

**Figure 8:**
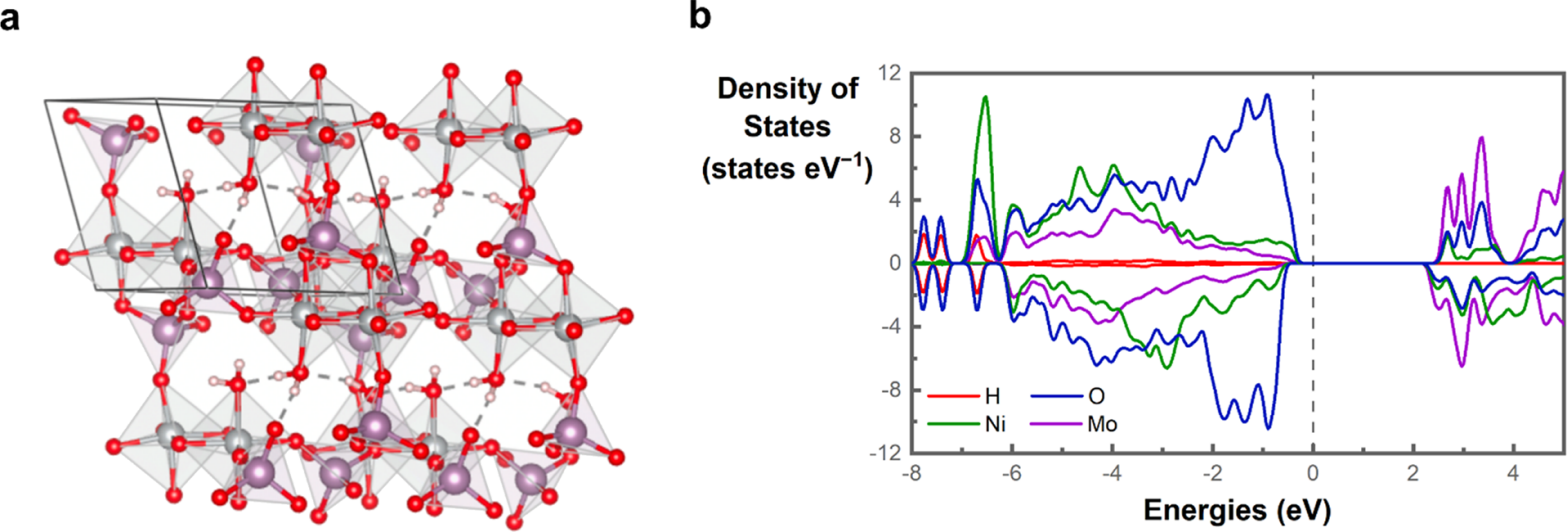
(a) Crystal structure of refined NiMoO_4_·3/4H_2_O with optimized water position and orientation forming hydrogen bonds. (b) The projected density of states for different elements in the majority and minority spin channels. Here, the computed band gap for the NiMoO_4_·3/4H_2_O under the considered level of theory is 2.19 eV, whereas the magnetization per Ni atoms is 1.8 μ_B_. It is important to emphasize that the performed calculations employed a ferromagnetic coupling to minimize the computational time associated with the distinct possible configurations of an antiferromagnetic state.

Based on Raman spectroscopy of the NMO-H_2_O-rods exhibiting two strong peaks at 949 and 958 cm^–2^, which are reported for molybdenum tetrahedrally and octahedrally coordinated by oxygen, respectively, and the Ni 2p high-resolution XPS (Figure S5b) as well as ZFC-FC indicating the presence of different nickel environments, one might hypothesize for the rod compound a highly disordered crystal structure with nickel partially on the molybdenum tetrahedral sites and *vice versa*. This hypothesis was investigated using the framework of density functional theory (DFT) together with a global structural energy minimization algorithm using minima hopping.^50^ The method utilizes local optimization and molecular dynamics to investigate the energy landscape of systems. With this, we can estimate the position of the water molecules within the crystal structure. Water molecules were initially distributed into the lattice in three different ways. The global energy ground state structural configuration was selected and analyzed. Based on the obtained ground state structure, we first investigated the formation of antisite defects (meaning the exchange of Ni atoms per Mo atoms). New global optimizations were performed considering the defective lattices, and their energy is at least 2.66 eV higher than the energy of the nondefective structure. Therefore, the formation of antisite defects is an unlikely process. The calculations also reveal that water molecules arrange in such a way that hydrogen bonds are formed inside the NiMoO_4_ · 3/4 H_2_O crystal lattice, where some Ni octahedra are coordinated by oxygen from the water molecule. Upon loss of coordination, water molecules are seen to bond to the central Ni, completing the octahedra. A representative structure is shown in Figure 8a, with the projected density of states (pDOS) that provides insights into the electronic structure of this material (Figure 8b). Here, one can note that O 2p dominates states close to the Fermi energy, indicating a high probability of defects in its anionic sublattice. O 2p band centers scale with the formation of oxygen defects and thermal stability of oxides.^82^ Moreover, reaction intermediates with an electron-withdrawing character will directly interact with the O anionic sublattice, likely leading to structural disorder due to oxygen defect formation.^83^

**Figure 9:**
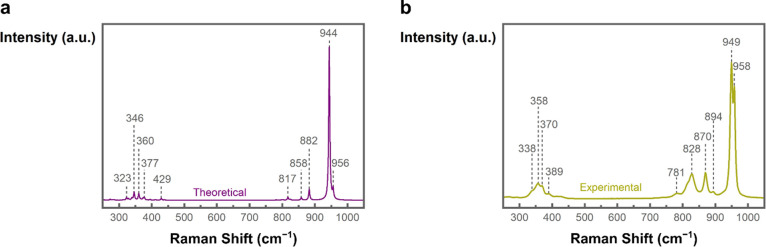
(a) Theoretical Raman spectra of the DFT refined structure with water coordination. (b) The corresponding experimental Raman spectra of the compound in the 3D NMO-H_2_O-rods.

To faithfully compare this situation with the experimental data, we performed DFT calculations to obtain the theoretical Raman spectrum of the optimized structure depicted in Figure 9a. An excellent match with the experimental Raman spectrum in Figure 9b is obtained, with several small intensity modes between the 300 and 400 cm^–1^ region, three modes in the 800–900 cm^–1^ region, and two modes in the 950 cm^–1^ region. As the Raman vibrations are intimately related to the forces between the nuclei and in turn dependent on the second derivative of their position, a matching Raman spectrum is a strong indication of improved structural accuracy.

## Conclusions

Two different phases of nickel molybdate hydrates, preferably structured into 2D nanosheets and 3D nanorods, are reported. The absence of nanosheet structures among the rods and *vice versa* was strongly suggested by electron microscopy as well as PXRD and Raman spectroscopy before and after selective molybdenum leaching and indicates that a controlled synthesis of both compounds and resulting nanostructures can be obtained. The presence of two different compounds in the two nanostructures was confirmed by EDX, XPS, ToF-ERDA, and RBS, which in collaboration with TGA proposed stoichiometries of NiMoO_4_·0.6H_2_O for 3D NMO-H_2_O-rods and approximately Ni_3_MoO_5–*x*_(OH)_*x*_·(2.3 – 0.5*x*)H_2_O for the 2D NMO-H_2_O-sheets. The NMO-H_2_O-rod materials seem to consist mainly of large 3D monocrystalline rods. The proposed triclinic crystal structure with space group *P*–1 is compatible, but the reported PDF 04-017-0338 seems to be not fully accurate. The Curie–Weiss analysis of ZFC-FC curves revealed a magnetic moment typical of Ni^2+^ in an octahedral ligand field in the rods. Theoretical assessment of the structure, where water comes in and coordinates the central Ni cation, could resolve the structure and faithfully reproduce the experimental Raman spectrum. In stark contrast, the NMO-H_2_O-sheets likely formed a layered structure, with small crystalline and amorphous regions building up each nearly 2D sheet. The magnetization and coordination analyses for the 2D sheets were more challenging but were compatible with a mixed valence Ni and thus a possible charge compensation effect and potential local 2D bonding, but with large uncertainties.

In summary, the two nanostructures are not polymorphs of the same hydrate material but fundamentally different compounds as revealed by elemental analysis and diffraction techniques. Vibrational spectroscopy and magnetization data revealed a different local environment around Ni and Mo. We would like to emphasize that because of the low intensity of the 2D nanosheet signal in PXRD and Raman spectra, it might often remain undetected in the simultaneous presence of the 3D (rod) structure. A promising method to probe its presence is the removal of molybdenum from the hydrate nanorod structure by immersing the material in 1 M KOH combined with, for example, Raman spectroscopy. The strong signal of the nanorod’s Mo–O vibration vanishes during this leaching process, and the signal of the stable nanosheet Mo–O vibration becomes visible. Despite our best effort and employment of several characterization techniques, we could not fully resolve the crystal structure of 3D NMO-H_2_O-rods. We can confirm that the proposed structure in PDF 04-017-0338 is not fully correct and that a disordered structure with nickel and molybdenum switching places is unlikely but the optimized water coordination forming a hydrogen bond network in the crystal structure led to an improved structure. Single-crystal XRD of a sufficiently large 3D NMO-H_2_O-rods crystal as well as X-ray absorption spectroscopy (XAS) could potentially resolve this uncertainty. In addition, XAS could also shed light on the oxidation states of nickel and molybdenum in the 2D NMO-H_2_O-sheets.

Discrimination between the two different nickel molybdate hydrate nanostructured compounds is anticipated to be of importance for understanding of the phase evolution under synthesis as well as when assessing structure-to-property relations in electrocatalytic applications, with catalysts derived from NMO-H_2_O nanostructures such as certain NiFe-LDH or Ni_4_Mo. We emphasize that when comparing nickel molybdates with (or derived from) differently shaped nanostructures, in fact, different compounds are compared and not only different nanostructures, which also complicate attributions regarding activity or stability in the case of both compounds present. To resolve uncertainties regarding said activity and stability, the present compounds or precatalysts (rod or sheet compound) have to be made clear, and their presence needs to be verified. To fully control the synthesis and establish a true material-to-property relationship, we believe that the work and included considerations would be helpful for the community, extending beyond the realm of 2D and 3D structured Ni-based molybdates. The findings and approach of this research could also have relevance for other hydrated metal-oxide-based systems, where enhancement of one of the elements may occur during hydrate-mediated formation and reconstruction.

## Supporting Information Available

Short overview of the utilized characterization methods for the different materials and their main findings; detailed description of synthesis and characterization techniques used; and additional SEM, EDX XPS, ToF-ERDA (including calculation of ion beam induced hydrogen loss calculation), RBS, TGA (including calculation of elemental composition), ZFC-FC, TEM, Raman spectroscopy, and FTIR of hydrate structures (PDF)
The cRED pattern of NMO-H_2_O-rods (MP4)
