# Supplementary material for: Clearing Up Discrepancies in 2D and 3D Nickel Molybdate Hydrate Structures
Source: Inorg Chem. 2024 Jan 19;63(5):2388–400. doi: 10.1021/acs.inorgchem.3c03261 (PMC10848204; doi:10.1021/acs.inorgchem.3c03261)
Supplement: Supplementary file 1 — ic3c03261_si_001.pdf [file ic3c03261_si_001.pdf]

Supporting Information for

Clearing Up Discrepancies in 2D and 3D Nickel Molybdate Hydrate Structures

*Robin N. Dürr,[a, b] Pierfrancesco Maltoni,[c] Shihui Feng,[d] Sagar Ghorai,[c] Petter Ström,[e] Cheuk-Wai Tai,[d] Rafael Barros Neves de Araujo,[c] and Tomas Edvinsson[c, f] **

[a] Department of Chemistry, Physical Chemistry, Ångström Laboratory, Uppsala University, 751 20 Uppsala, Sweden

[b] Université Paris-Saclay, CEA, CNRS, NIMBE, LICSEN, 91191 Gif-sur-Yvette, France

[c] Department of Materials Science and Engineering, Solid State Physics, Ångström Laboratory, Uppsala University, 751 03 Uppsala, Sweden

[d] Department of Materials and Environmental Chemistry, Stockholm University, 106 91 Stockholm, Sweden

[e] Department of Physics and Astronomy, Applied Nuclear Physics, Ångström Laboratory, Uppsala University, 751 20 Uppsala, Sweden

[f] Energy Materials Laboratory, Chemistry: School of Natural and Environmental Science, Newcastle University, Newcastle Upon Tyne NE1 7RU, United Kingdom

* Corresponding author: tomas.edvinsson@angstrom.uu.se

Contents

Experimental 4
Scanning Electron Microscopy (SEM) of NMO-H_2O nanostructures 7
Energy Dispersive X-ray Spectroscopy (EDX) of NMO-H_2O nanostructures 9
X-ray Photoelectron Spectroscopy (XPS) of NMO-H_2O nanostructures 11
Ion Beam Analysis (IBA) of NMO-H_2O nanostructures 14
Thermogravimetric Analysis (TGA) of NMO-H_2O nanostructures 16
Zero Field Cooled (ZFC) and Field Cooled (FC) measurement of NMO-H_2O nanostructures ... 22
Transmission Electron Microscopy (TEM) of NMO-H_2O nanostructures 25
Raman Spectroscopy of NMO-H_2O nanostructures 28
Fourier Transform Infrared Spectroscopy (FTIR) of NMO-H_2O nanostructures 34
Bibliography 37

Chart S1. Overview of the synthesis of the different samples and the used characterization methods with the main findings. Abbreviations are explained in the main text.

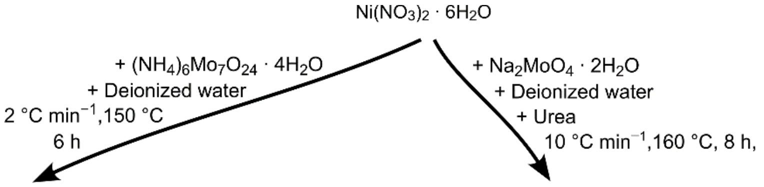

NMO-H_2O-rods

SEM, EDX: *Rod shape, homogeneous dispersion of Ni, Mo, and O.*
TEM, SAED: *Monocrystallinity of rods. No plane was found fitting to the proposed crystal structure by PDF 04-017-0338.*
3D ED: *Triclinic system with P-1 space group. Refinement differs from PDF 04-017-0338.*
XPS: *Mo in +VI oxidation state.*
IBA: *Elemental concentration of Ni, Mo, O, (and H).*
TGA: *Calculation of chemical composition ($NiMoO_4 \cdot 0.6H_2O$).*
ZFC-FC: *Paramagnetic to antiferromagnetic transition. Ni in oxidation state +II.*
PXRD: *3D crystalline. Proposed crystal structure from PDF 04-017-0338 is close, but not perfect.*
Raman: *Raman bands different than for NMO-H_2O-sheets.*
Selective Mo leaching: *Chemical instability causes leaching of Mo from the structure. No NMO-H_2O-sheets present.*
ATR-FTIR: *Different spectrum than for NMO-H_2O-sheets.*
DFT: *Exchange of Ni and Mo unlikely. Crystal water forms hydrogen bond network. Theoretical Raman spectrum of refined structure close to experimental.*

NMO-H_2O-sheets

SEM, EDX: *Sheet shape, homogeneous dispersion of Ni, Mo, and O.*
TEM, SAED: *Thickness of sheets. Polycrystalline with amorphous regions.*
XPS: *Mo in +V and +VI oxidation state.*
IBA: *Elemental concentration of Ni, Mo, O, (and H).*
TGA: *Calculation of chemical composition ($Ni_3MoO_5 \cdot 2.3H_2O$).*
ZFC-FC: *(Ferromagnetism) and magnetic ordering.*
PXRD: *2D crystalline, possibly layered structure. No NMO-H_2O-rods present.*
Raman: *Raman bands differ from NMO-H_2O-rods. No NMO-H_2O-rods present.*
Selective Mo leaching: *Not affected.*
ATR-FTIR: *Different spectrum than for NMO-H_2O-rods.*

Experimental

Synthesis

Nickel molybdate hydrate nanorods (NMO-H_2O-rods) were synthesized in a hydrothermal synthesis. In a representative synthesis 0.01 M $(NH_4)_6Mo_7O_{24} \cdot 4H_2O$ (AHM) and 0.07 M $Ni(NO_3)_2 \cdot 6H_2O$ was dissolved in 60 mL deionized water (DI). These concentrations were chosen to provide a 1:1 molar ratio between molybdenum and nickel, as for the 2D nanosheet synthesis below. The solution was transferred into a 100 mL Teflon lined stainless steel autoclave and heated up in a muffle furnace to 150 °C with 2 °C min^{-1} and hold at this temperature for 6 h. After cooling to room temperature, the yellow precipitate was washed with DI three times followed by one time with ethanol, collected each time by centrifugation and finally dried at 60 °C. It should be noted that the reaction solution after the synthesis was still clear green. In order to remove lattice water, the dry precipitate was further hold at 200 °C for 8 h.

For nickel molybdate hydrate nanosheets (NMO-H_2O-sheets) the synthesis route of Chen *et al.* was mostly followed. [1] Herein 2 mmol $Na_2MoO_4 \cdot 2H_2O$ and 2 mmol $Ni(NO_3)_2 \cdot 6H_2O$ was dissolved in 60 mL (DI). Then 8 mmol urea was added. After stirring for 30 minutes the solution was transferred into a 100 mL Teflon lined stainless steel autoclave and heated up in a muffle furnace to 160 °C and hold for 8 h. Since no heating ramp was reported in the original paper, a rate of 10 °C min^{-1} was applied. After synthesis the solution was clear and colorless with bright green precipitate. Also, those precipitates were washed, collected and dried as the NMO-H_2O-rods above.

Characterization

Scanning Electron Microscopy (SEM) and *Energy Dispersive X-ray Spectroscopy (EDX).* The nanostructures were analyzed with a high-resolution SEM (ZEISS 1530) with a Schottky field emission gun and acceleration voltages of 15 - 20 kV. The secondary electrons were detected with an Inlens detectors. The software used was SmartSEM (Version 5.07). EDX were detected with an Oxford Instruments X-Max^N detector. The software AZTec was used for acquiring and analyzing the detected X-rays.

X-Ray Photoelectron Spectroscopy (XPS). XPS analysis was performed with a Physical Electronics PHI Quantera II Scanning XPS Microprobe. Monochromatic Al Kα X-rays with 1486.6 eV and acceleration voltage of 15 kV were used. For all survey spectra the sample was illuminated with a 200 µm diameter X-ray beam with a power of 50 W. A pass energy of 224.00 eV with an acquisition time of 50 ms per step and a step size of 0.8 eV. For the high-resolution spectra, an X-ray beam diameter of 100 µm was used with a pass energy of 55.00 eV, an acquisition time of 50 ms per step and a step size of 0.1 eV. For the NMO-H_2O-rods the acquisition on Ni, Mo, O and C was repeated 20, 10, 10 and 10 times, respectively. For the NMO-H_2O-sheets the acquisition on Ni, Mo, O and C was repeated 10, 20, 10 and 10 times, respectively. All spectra were analyzed

with CasaXPS software (Version 2.3.24).[2] For elemental analysis a Shirley background correction was used. Prior to analysis, all spectra were charge corrected versus adventitious carbon at 284.8 eV binding energy.

Powder X-ray Diffraction (PXRD). PXRD was done using a Bruker D8 Advance diffractometer with a Cu Kα radiation in a Bragg-Brentano geometry and a solid state rapid LynxEye detector. The software used was DIFFRACT plus software and the patterns acquired at room temperature between 5° - 90° 2θ with at step size of 0.013° and a time per step of 4 seconds. The collected data were analyzed using HighScore Plus 3.0 software from PANalytical.

Transmission Electron Microscopy (TEM). TEM analysis was conducted with a JEOL JEM-2100F with a Schottky-type field emission gun and an acceleration voltage of 200 kV. Bright Field images and Selected Area Electron Diffraction patterns were taken with a Gatan Ultrascan 100 and Orius 200 D camera, respectively. The software for acquisition was Gatan Microscopy Suite while for analysis ImageJ (Version 1.53) was used. ImageJ was used for post processing the TEM images.[3] For three-dimensional electron diffraction (3D ED) the single crystal samples were first separated by ultrasonication in ethanol. The solution was drop casted on a copper TEM grid and analyzed in a ThermoFisher Themis Z microscope operating with an acceleration voltage of 300 kV. The diffraction pattern was detected with a Gatan Oneview camera. A camera length of 360 mm and a spot size of 3 were used. A self-developed DigitalMicrograph plugin was used to control the data collection.[4] Diffraction data were acquired by continuously rotating the sample stage with the crystal at a rate of 1.47 ° s^{-1}. The acquisition time was set to 0.125 s, meaning the individual diffraction images were integrated over 0.18 ° of reciprocal space. Several data sets from different single crystals were collected by 110 ° rotation angle. For the analysis the cRED processing software REDp was utilized to determine the unit cell parameters and the space group based on the symmetry and reflection conditions of the collected diffraction pattern.[5] The data were processed with the crystallography software XDS.[6] The obtained data sets were merged together according to their cross correlation in order to improve the level of completion and to obtain a single data set suitable for structure solution and refinement. For solving the structure according to their lowest cost function, the software SHELXT and SHELXLE were used.[7,8] For illustration of the crystal structure VESTA Version 3.5.2 was used.[9]

Zero Field Cooled and Field Cooled (ZFC-FC) and Isothermal Field-dependent Magnetization Loops. Magnetization measurements were carried out using a MPMS XL SQUID magnetometer equipped with a superconducting magnet (up to ±50 kOe) from Quantum Design, Inc. The sample was immobilized in parafilm in a capsule to prevent any movement of the particles during the measurements. The thermal dependence of magnetization was measured according to the zero-field cooled (ZFC) and field cooled (FC) protocols. ZFC and FC measurements were carried out as follows: first, the sample was cooled down from 300 K to 5 K with no magnetic field applied. Then

a static magnetic field of 100 Oe was applied and the magnetization was measured during warming up from 5 K to 300 K. Finally, the sample was cooled down to 5 K under the same magnetic field and the magnetization was measured during the cooling. Isothermal field-dependent magnetization loops were recorded at 5 K and 300 K by sweeping the field between −50 kOe and 50 kOe. The obtained magnetization values were normalized by the weight of the powder present in the sample (≈ 10 mg).

Rutherford Backscattering Spectrometry (RBS). For RBS a 2 MeV $^{4}He^{+}$ beam was hitting the sample at an incident angle of 5 ° with respect to the surface normal. The sample was a pellet (d = 7 mm) of the of the NMO-H_2O nanostructures powder compressed with approximately 2 t. A passivated implanted planar silicon (PIPS) energy detector for scattered particles was placed at a backscattering angle of 170°. Outgoing particle trajectories were inclined 5 ° with respect to the sample surface normal. The analysis was conducted with the software Simnra (Version 7.03).[10]

Time-of-Flight Elastic Recoil Detection Analysis (ToF-ERDA). For ToF-ERDA a beam of 44 MeV $^{127}I^{10+}$ ions were used to bombard the compressed pellet previously analyzed with RBS. The incident beam hit the sample at an angle of (23 ± 1)° to the sample surface. The detector was positioned at an angle of 45 ° with regard to the forward beam direction.[11] For detection an electron mirror flight time detector was employed together with an energy resolving gas ionization chamber. For the data analysis the software Potku, developed at the University of Jyväskylä, was employed.[12]

Thermogravimetric Analysis (TGA). TGA was conducted with a TA Instruments TGA Q500 instrument in air from room temperature to 600 °C with 2 °C min^{-1} on a platinum pan. Data were collected and analyzed with Universal Analysis 2000 (Version 4.5A).

Raman Spectroscopy. Most Raman spectra were acquired with Renishaw Reflex (Invia) Raman spectrometer. In this spectrometer a frequency doubled Nd:YAG 532 nm laser with a grating of 2400 lines mm^{-1} and a Renishaw streamline CCD 1024 chip detector were used. The software used for acquisition was WiRE 3.4. For the analysis with 785 nm laser excitation a Renishaw Qontor (Invia) Raman Spectrometer with the Renishaw HPNIR 785 semiconductor laser source, a grating of 1200 lines mm^{-1} and a Centrus detector was employed. The software used was WiRE 5.5. Prior to all experiments, the Raman spectrometer was calibrated with a silicon reference to (520.5 ± 0.2) cm^{-1}. All spectra were taken with a 20× magnification lens.

Attenuated Total Reflection Fourier-Transform Infrared Spectroscopy (ATR-FTIR). ATR-FTIR was conducted with a Bruker Vertex 70v spectrometer with a A225/Q Platinum ATR diamond unit. For the background and sample 16 scans were acquired from 4500 cm^{-1} to 200 cm^{-1} with a resolution of 2 cm^{-1}. The software used for acquisition was Opus (Version 8.2.21).

Scanning Electron Microscopy (SEM) of NMO-H_2O nanostructures

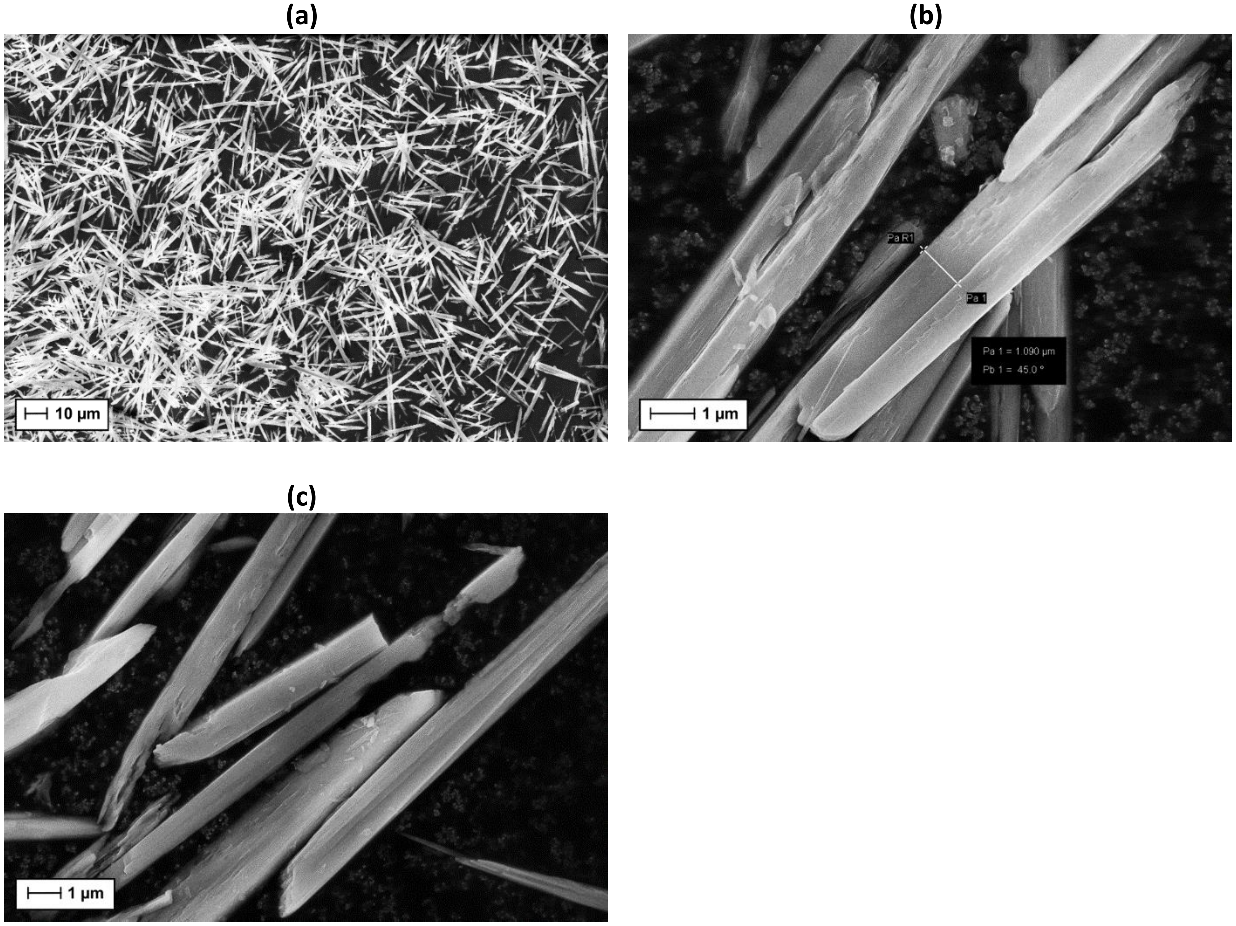

Figure S1. Secondary electron (SE) images of NMO-H_2O-rods with different resolutions confirming the synthesis of nanorod shaped structures.

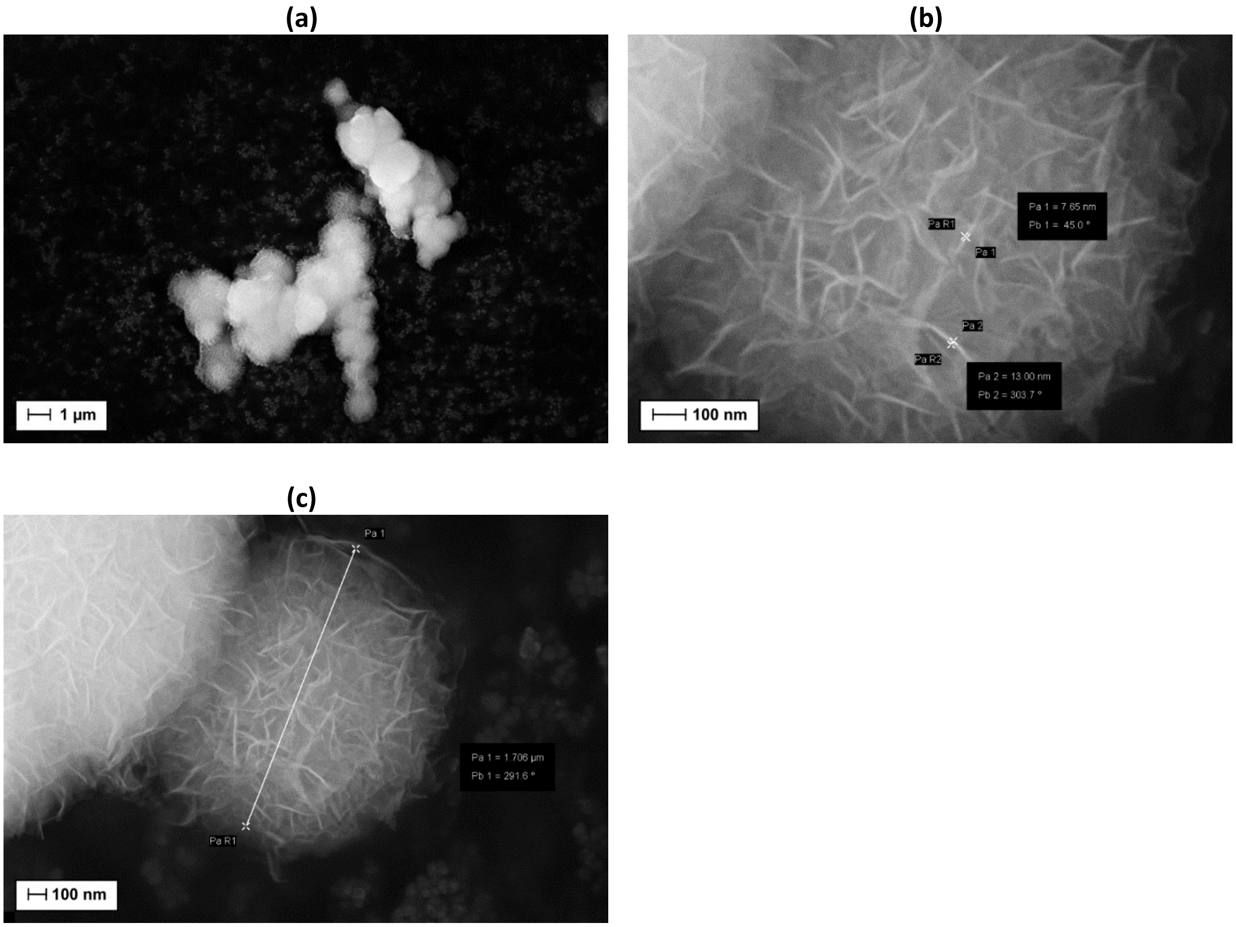

Figure S2. SE images of NMO-H_2O-sheets with different resolutions confirming the synthesis of nanosheet shaped structures.

Energy Dispersive X-ray Spectroscopy (EDX) of NMO-H_2O nanostructures

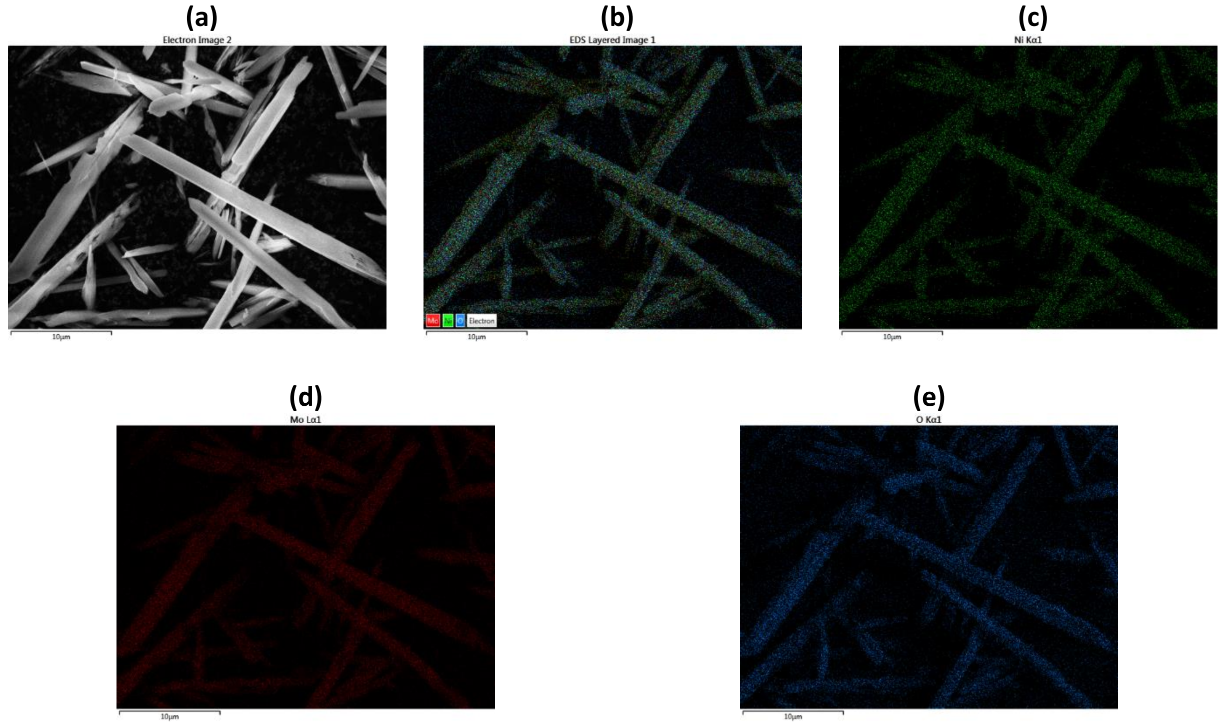

Figure S3. Energy dispersive x-ray spectroscopy (EDX) mapping of NMO-H_2O-rods. **(a)** SE of the area of interest; **(b)** Overlay of SE image, Ni, Mo, and O mapping; **(c)** Ni mapping; **(d)** Mo mapping; **(e)** O mapping.

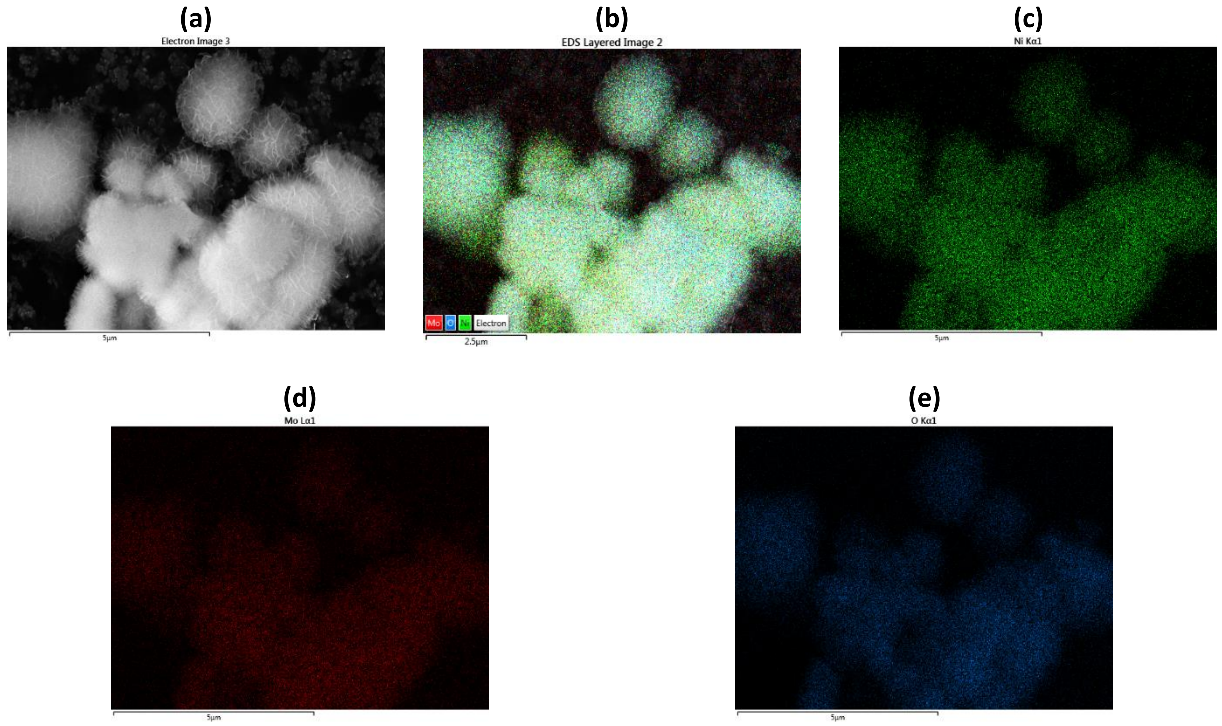

Figure S4. EDX mapping of NMO-H_2O-sheets. **(a)** SE of the region of interest; **(b)** Overlay of SE image, Ni, Mo, and O mapping; **(c)** Ni mapping; **(d)** Mo mapping; **(e)** O mapping.

X-ray Photoelectron Spectroscopy (XPS) of NMO-H_2O nanostructures

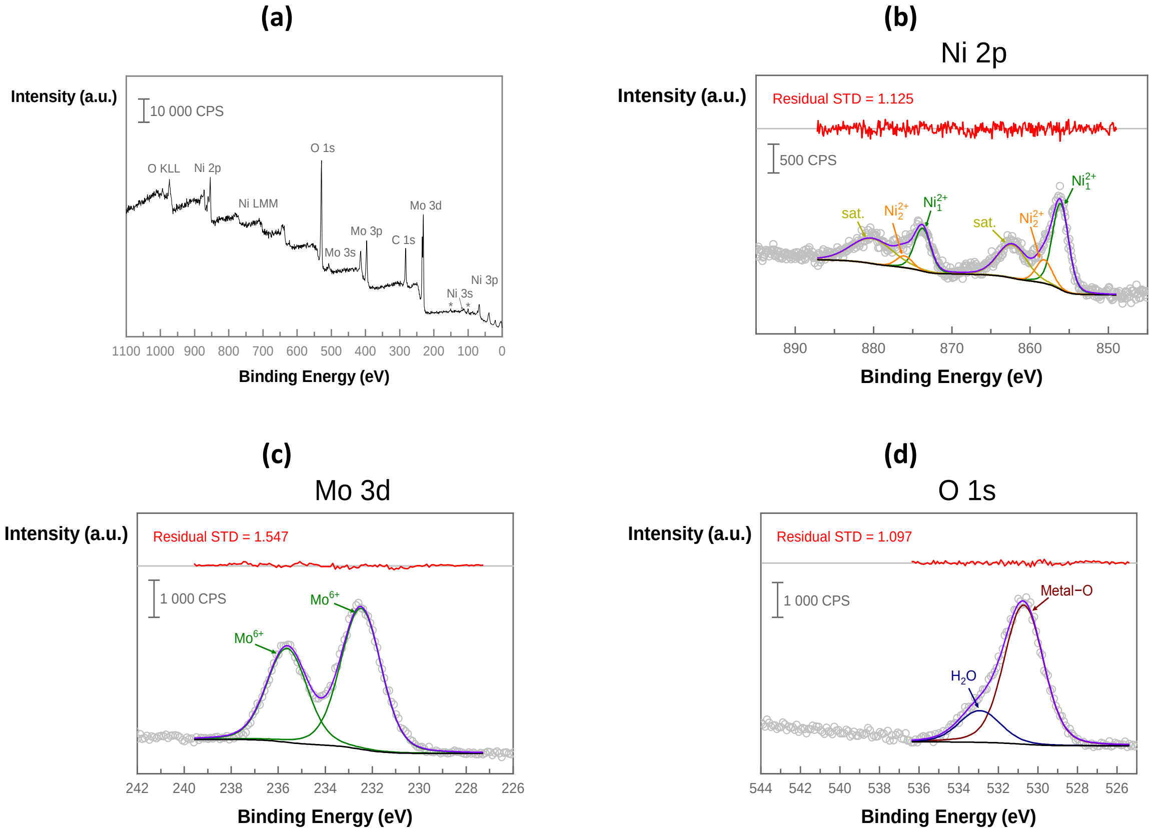

Figure S5. XPS analysis of NMO-H_2O-rods. **(a)** Survey scan; **(b)** high-resolution Ni 2p spectrum; **(c)** high-resolution Mo 3d spectrum; **(d)** high-resolution O 1s spectrum.

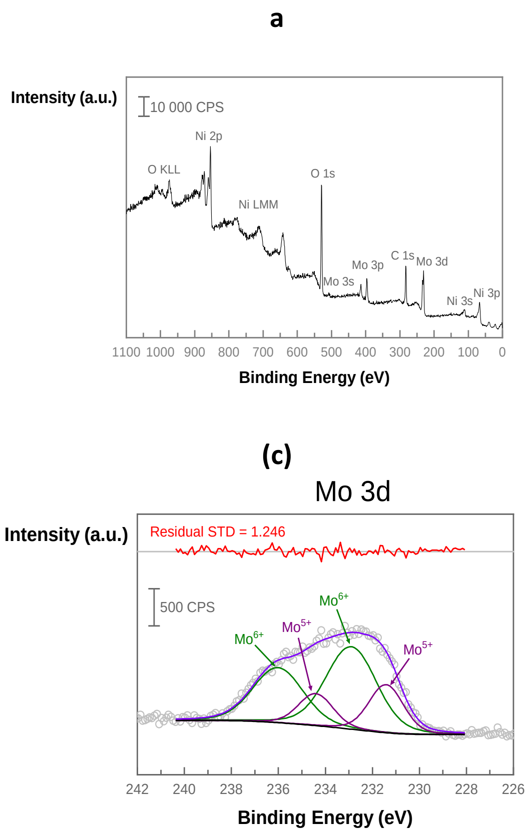

Figure S6. XPS analysis of NMO-H_2O-sheets. **(a)** Survey scan; **(b)** high-resolution Ni 2p spectrum; **(c)** high-resolution Mo 3d spectrum; **(d)** high-resolution O 1s spectrum.

Table S1. Quantification of NMO-H_2O nanostructures based on high-resolution XPS.

Sample	Orbital	Position	FWHM	RSF	TF	Area	Area/(RSF×TF ×MFP)	At. %	Corr. At. %
NMO-H_2O-rods	Mo 3d	232.58	2.13	4.0307	48.829	14222	72.26	14.91	17.37
	Ni 2p	856.18	2.36	4.0574	52.89	13922	64.88	13.39	15.60
	O 1s	530.58	2.48	0.733	50.819	12938	347.34	71.69	67.03
NMO-H_2O-sheets	Mo 3d	233.16	5.36	4.0307	48.83	7283.6	37.01	7.73	9.68
	Ni 2p	856.46	3.78	4.0574	52.889	24951	116.27	24.28	30.41
	O 1s	530.76	3.28	0.733	50.818	12131	325.67	68.00	59.90

FWHM: Full width half maximum

RSF: Relative sensitivity factor

TF: Transmission function

MFP: Mean free path

In the last column labeled "Corr. At.%", the likely oxygen contamination from the Leit tab was compensated, assuming all detected carbon originated from the Leit tab. Previous measurements have shown that without the Leit tab there is only a very small amount of adventitious carbon present, so the real oxygen content may be in between the presented ones.

Ion Beam Analysis (IBA) of NMO-H_2O nanostructures

(a) **(b)**

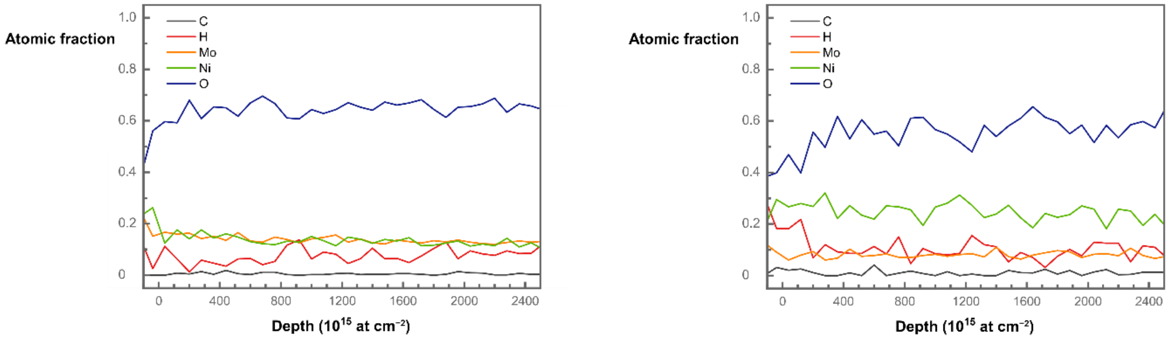

Figure S7. ToF-ERDA depth profiling of each element in **(a)** NMO-H_2O-rods and **(b)** NMO-H_2O-sheets. Both depth profiles are normalized at each depth.

Depth channels between 800 – 2000 × 10^{15} at. cm^{-2} were used to obtain the composition of the bulk material.

(a) **(b)**

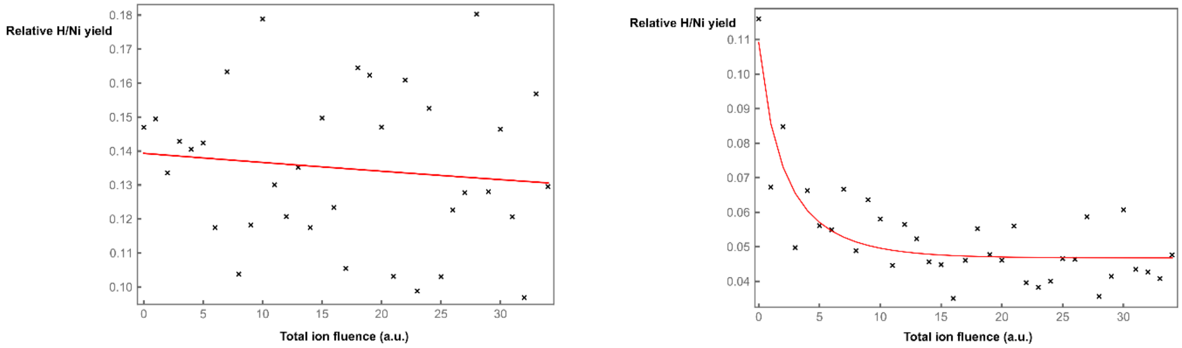

Figure S8. Fit of the relative H/Ni yield over the total ion fluence. **(a)** NMO-H_2O-rods and **(b)** NMO-H_2O-sheets. The graph for the sheets shows a clear ion beam induced hydrogen loss.

The H/Ni ratio shows a clear decay over the total ion fluence for NMO-H_2O-sheets. The datapoints were fitted according to following expression [13]

$$\rho(x) = \left(\frac{1}{\rho_f} + \left(\frac{1}{\rho_0} - \frac{1}{\rho_f}\right) \times e^{-Kx}\right)^{-1} \quad \text{(S1)}$$

with $\rho(x)$ being the hydrogen to nickel ratio at the total ion fluence x, ρ_o and ρ_f the initial and final hydrogen to nickel ratio, respectively and K the decay constant. The compensation factor for the hydrogen content is calculated with [14]

$$q = \rho_0 \times \overline{\rho(x)}^{-1} \quad \text{(S2)}$$

with q being the compensation factor for the hydrogen content and $\overline{\rho(x)}$ the average hydrogen to nickel ratio over the whole ion fluence. The atomic concentrations detected by ToF-ERDA depth profile are corrected before the nickel to molybdenum ratio refinement by RBS.

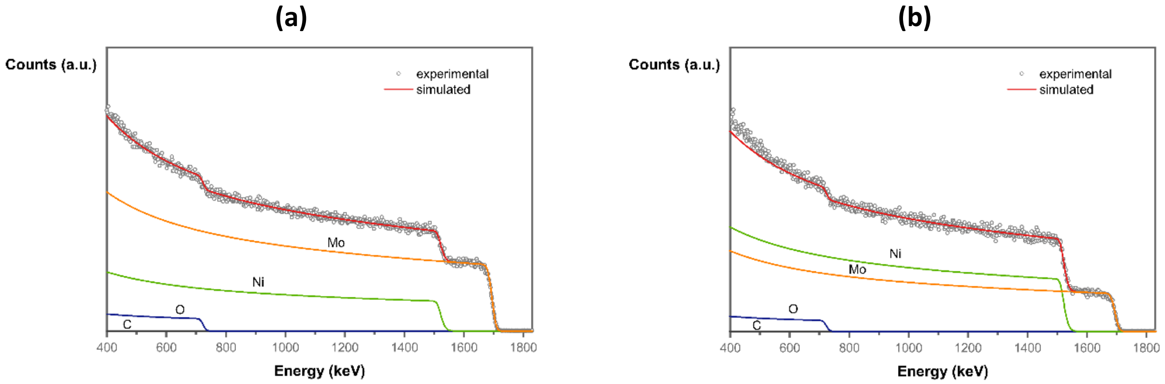

Figure S9. RBS analysis of **(a)** NMO-H_2O-rods and **(b)** NMO-H_2O-sheets.

Thermogravimetric Analysis (TGA) of NMO-H_2O nanostructures

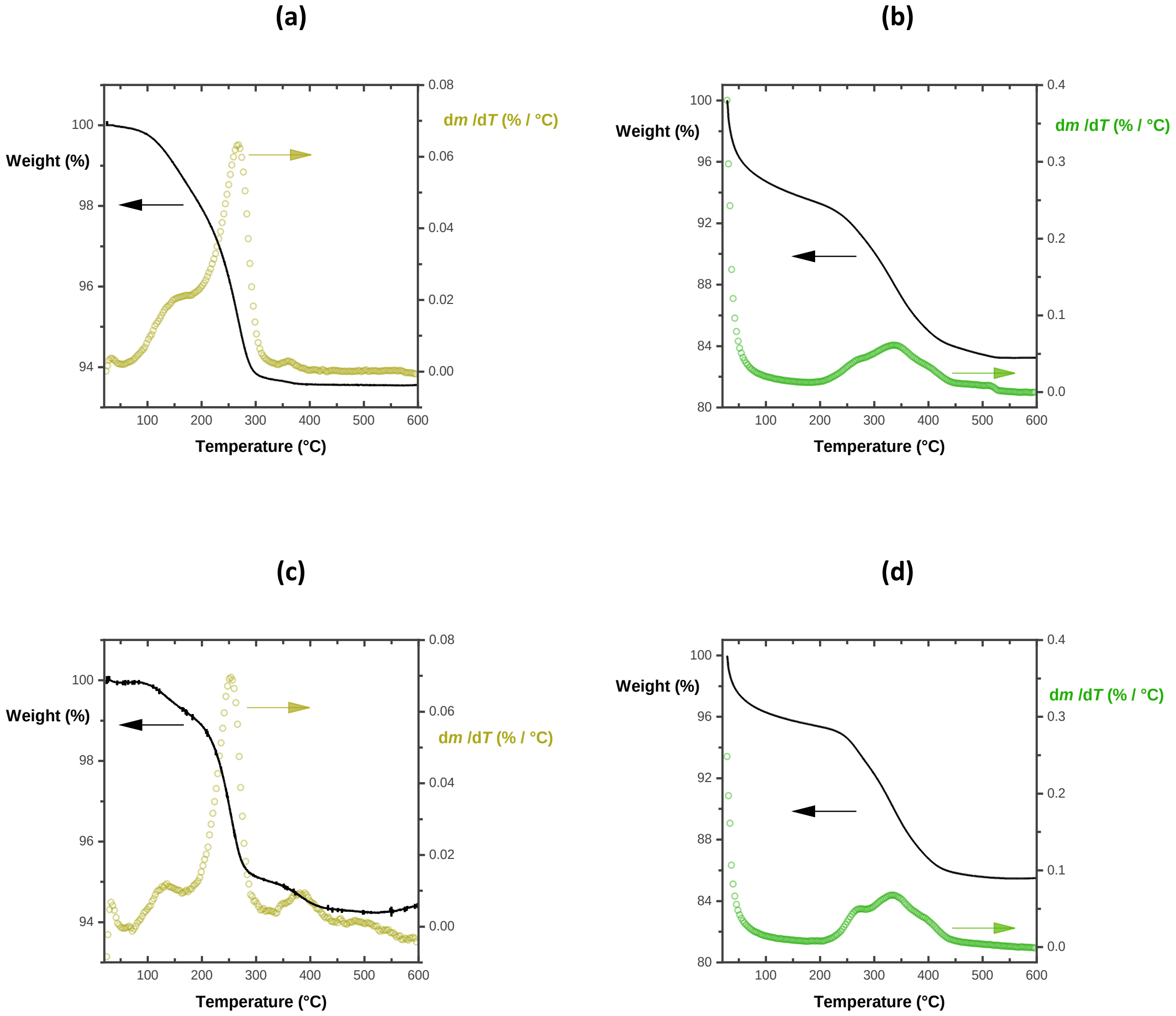

Figure S10. TGA analysis from 20 – 600 °C in air (2 °C min^{-1} ramp) with weight loss on left y-axis and differential of weight loss over temperature on right y-axis for **(a)** NMO-H_2O-rods before heating at 200 °C; **(b)** NMO-H_2O-sheets before heating at 200 °C; **(c)** NMO-H_2O-rods after heating at 200 °C; **(d)** NMO-H_2O-sheets after heating at 200 °C.

Note S1. Calculation of chemical formula based on TGA and IBA

The amount of water present is calculated with the following equation, here shown for the temperature range 20 – 550 °C:

$$wt.\%_{550\,°C} = \frac{n_{\mathrm{Ni}} \times M_{\mathrm{Ni}} + n_{\mathrm{Mo}} \times M_{\mathrm{Mo}} + n_{\sum \mathrm{O}} \times M_{\mathrm{O}} - n_{\mathrm{H2O}} \times M_O}{n_{\mathrm{Ni}} \times M_{\mathrm{Ni}} + n_{\mathrm{Mo}} \times M_{\mathrm{Mo}} + n_{\sum \mathrm{O}} \times M_{\mathrm{O}} + n_{\mathrm{H2O}} \times 2 \times M_H} \quad \text{(S3)}$$

Atomic masses are taken from the periodic system of elements:

M_{Ni} = 58.693

M_{Mo} = 95.950

M_{O} = 15.999

M_{H} = 1.008

Calculation for NMO-H_2O-rods:

The initial amount of substance is taken from IBA.

$n_{\mathrm{Ni}} = 0.1344$

$n_{\mathrm{Mo}} = 0.1356$

$n_{\sum \mathrm{O}} = 0.6458$

$n_{\mathrm{H2O}} =$ variable solved for

The loss of mass is taken from TGA:

Weight percent at 550 °C: 94.29 wt.%

Weight percent at 600 °C: 94.41 wt.%

The equation was solved with WolframAlpha.[15]

The amount of water present was calculated as $n_{\mathrm{H2O}} = 0.0996$ and $n_{\mathrm{H2O}} = 0.0975$ for using the remaining weight percent at 550 °C and 600 °C, respectively. Since the concentration of water is known, the amount of oxygen in oxide is also known ($n_{\mathrm{O}} = n_{\sum \mathrm{O}} - n_{\mathrm{H2O}}$). Furthermore, water is expected in the form of crystal water (CW) and reversibly bond water (RW) following the equation:

$$n_{\mathrm{H2O}} = n_{\mathrm{CW}} + n_{\mathrm{RW}} \tag{S4}$$

And hence

$$n_{\sum \mathrm{O}} = n_{\mathrm{O}} + n_{\mathrm{CW}} + n_{\mathrm{RW}} \tag{S5}$$

The amount of reversibly bond water is calculated the following way:

$$wt.\% = \frac{n_{\mathrm{Ni}} \times M_{\mathrm{Ni}} + n_{\mathrm{Mo}} \times M_{\mathrm{Mo}} + (n_{\sum \mathrm{O}} - n_{\mathrm{RW}}) \times M_{\mathrm{O}} + (n_{\mathrm{H2O}} - n_{\mathrm{RW}}) \times 2 \times M_{H}}{n_{\mathrm{Ni}} \times M_{\mathrm{Ni}} + n_{\mathrm{Mo}} \times M_{\mathrm{Mo}} + n_{\sum \mathrm{O}} \times M_{\mathrm{O}} + n_{\mathrm{H2O}} \times 2 \times M_{H}} \tag{S6}$$

To estimate the amount of reversibly bond water two temperatures at the onset of the removal of crystal water are chosen from TGA.

Weight percent at 180 °C: 99.12 wt.%

Weight percent at 200 °C: 98.87 wt.%

Solving the equation (S6) for n_{RW} gives the amount of reversibly bond water and subsequently of crystal water.

Table S2. Calculated amount of total water and its contribution of crystal water and reversibly bond water in NMO-H_2O-rods, normalized to molybdenum content. Two different temperatures for both, after removal of reversibly bond water (550 °C and 600 °C) and after removal of reversibly bond and crystal water (180 °C and 200 °C) is chosen.

$NiMoO_4$	Water	Crystal water	Reversibly bond water
20 °C – 550 °C	0.73		
20 °C – 180 °C		0.62	0.11
20 °C – 200 °C		0.59	0.15
20 °C – 600 °C	0.72		
20 °C – 180 °C		0.61	0.11
20 °C – 200 °C		0.57	0.15

To probe a possible removal of crystal water during the drying process at 200 °C for 8 hours TGA was conducted before and after drying. The percental loss of weight after removal of all water compared to before the removal of crystal water for the two NMO-H_2O-rods samples is (4.69 ± 0.13) % and (4.71 ± 0.21) % for the dried and not dried sample, respectively. This indicates that no significant amount of crystal water was removed during the drying process.

All the calculations above assumed that the IBA concentration represents NMO-H_2O with reversibly bond water. However, it is uncertain how much reversibly bond water was present during the analysis, hence another stoichiometry is calculated assuming no reversibly bond water in the IBA according to

$$\frac{wt.\%_{\mathrm{w.o.\ CW}}}{wt.\%_{\mathrm{w.\ CW}}} = \frac{n_{\mathrm{Ni}} \times M_{\mathrm{Ni}} + n_{\mathrm{Mo}} \times M_{\mathrm{Mo}} + (n_{\Sigma\,\mathrm{O}} - n_{\mathrm{CW}}) \times M_{\mathrm{O}}}{n_{\mathrm{Ni}} \times M_{\mathrm{Ni}} + n_{\mathrm{Mo}} \times M_{\mathrm{Mo}} + n_{\Sigma\,\mathrm{O}} \times M_{\mathrm{O}} + n_{\mathrm{CW}} \times 2 \times M_H} \tag{S7}$$

In which $wt.\%_{\mathrm{w.o.\ CW}}$ represents the measured weight percent after removal of crystal water (550 °C, 600 °C) and $wt.\%_{\mathrm{w.\ CW}}$ represents the measured weight percent with only crystal water (180 °C, 200 °C).

For NMO-H_2O-sheets an additional contribution of the possible presence of hydroxide has to be added.

Assuming that hydroxides are present and during the TGA dehydrate according to:

$$2\ \mathrm{OH}^- \xrightarrow{\Delta} \mathrm{O} + \mathrm{H_2O} \tag{S8}$$

Hence only 0.5 of the original oxygen content from the hydroxide is present after heating.

The equation for the calculation of evaporated water is modified to:

$$wt.\% = \frac{n_{\mathrm{Ni}} \times M_{\mathrm{Ni}} + n_{\mathrm{Mo}} \times M_{\mathrm{Mo}} + (n_{\mathrm{O}} + \frac{1}{2}\, n_{\mathrm{OH}}) \times M_{\mathrm{O}}}{n_{\mathrm{Ni}} \times M_{\mathrm{Ni}} + n_{\mathrm{Mo}} \times M_{\mathrm{Mo}} + (n_{\mathrm{O}} + n_{\mathrm{OH}} + n_{\mathrm{CW}} + n_{\mathrm{RW}}) \times M_O + (n_{\mathrm{OH}} + 2 \times n_{\mathrm{CW}} + 2 \times n_{\mathrm{RW}}) \times M_{\mathrm{H}}} \tag{S9}$$

With:

$$n_{\Sigma\,\mathrm{O}} = n_{\mathrm{O}} + n_{\mathrm{OH}} + n_{\mathrm{CW}} + n_{\mathrm{RW}} \tag{S10}$$

And:

$$n_{\mathrm{O,evap}} = \frac{1}{2} n_{\mathrm{OH}} + n_{\mathrm{CW}} + n_{\mathrm{RW}} \tag{S11}$$

Follows:

$$n_{\sum O} = n_{\mathrm{O}} + \frac{1}{2}n_{\mathrm{OH}} + \frac{1}{2}n_{\mathrm{OH}} + n_{\mathrm{CW}} + n_{\mathrm{RW}} = n_{\mathrm{O}} + \frac{1}{2}n_{\mathrm{OH}} + n_{\mathrm{O,evap}} \tag{S12}$$

Inserting (S11) and (S12) in (S9) yields:

$$wt.\% = \frac{n_{\mathrm{Ni}} \times M_{\mathrm{Ni}} + n_{\mathrm{Mo}} \times M_{\mathrm{Mo}} + (n_{\sum \mathrm{O}} - n_{\mathrm{O,evap}}) \times M_{\mathrm{O}}}{n_{\mathrm{Ni}} \times M_{\mathrm{Ni}} + n_{\mathrm{Mo}} \times M_{\mathrm{Mo}} + n_{\sum \mathrm{O}} \times M_{\mathrm{O}} + 2 \times n_{\mathrm{O,evap}} \times M_{\mathrm{H}}} \tag{S13}$$

This equation is used for calculating the $n_{\mathrm{O,evap}}$ with:

$n_{\mathrm{Ni}} = 0.2229$

$n_{\mathrm{Mo}} = 0.0736$

$n_{\sum \mathrm{O}} = 0.5381$

n_{O} = amount of oxygen in the oxide

n_{OH} = amount of oxygen in the hydroxide

n_{CW} = amount of oxygen in crystal water

n_{RW} = amount of oxygen in reversibly bond water

$n_{\mathrm{O,evap}}$ = variable solved for

$wt.\%$ = remaining weight at 550 °C/ 600 °C

With the known amount of evaporated oxygen, the amount of reversibly bond water can be calculated using the remaining weight at 180°C and 200 °C.

$$\begin{aligned} wt.\% &= \frac{n_{\mathrm{Ni}} \times M_{\mathrm{Ni}} + n_{\mathrm{Mo}} \times M_{\mathrm{Mo}} + n_{\mathrm{O}} \times M_{\mathrm{O}} + n_{\mathrm{OH}} \times M_{\mathrm{O}} + n_{\mathrm{OH}} \times M_{\mathrm{H}} + n_{\mathrm{CW}} \times M_{\mathrm{O}} + n_{\mathrm{CW}} \times 2 \times M_{\mathrm{H}}}{n_{\mathrm{Ni}} \times M_{\mathrm{Ni}} + n_{\mathrm{Mo}} \times M_{\mathrm{Mo}} + (n_{\mathrm{O}} + n_{\mathrm{OH}} + n_{\mathrm{CW}} + n_{\mathrm{RW}}) \times M_{\mathrm{O}} + (n_{\mathrm{OH}} + 2 \times n_{\mathrm{CW}} + 2 \times n_{\mathrm{RW}}) \times M_{\mathrm{H}}} \\ &= \frac{n_{\mathrm{Ni}} \times M_{\mathrm{Ni}} + n_{\mathrm{Mo}} \times M_{\mathrm{Mo}} + (n_{\mathrm{O}} + n_{\mathrm{OH}} + n_{\mathrm{CW}}) \times M_{\mathrm{O}} + 2 \times (\frac{1}{2} \times n_{\mathrm{OH}} + n_{\mathrm{CW}}) \times M_{\mathrm{H}}}{n_{\mathrm{Ni}} \times M_{\mathrm{Ni}} + n_{\mathrm{Mo}} \times M_{\mathrm{Mo}} + (n_{\mathrm{O}} + n_{\mathrm{OH}} + n_{\mathrm{CW}} + n_{\mathrm{RW}}) \times M_{\mathrm{O}} + (n_{\mathrm{OH}} + 2 \times n_{\mathrm{CW}} + 2 \times n_{\mathrm{RW}}) \times M_{\mathrm{H}}} \\ &= \frac{n_{\mathrm{Ni}} \times M_{\mathrm{Ni}} + n_{\mathrm{Mo}} \times M_{\mathrm{Mo}} + (n_{\sum \mathrm{O}} - n_{\mathrm{RW}}) \times M_{\mathrm{O}} + 2 \times (n_{\mathrm{O,evap}} - n_{\mathrm{RW}}) \times M_{\mathrm{H}}}{n_{\mathrm{Ni}} \times M_{\mathrm{Ni}} + n_{\mathrm{Mo}} \times M_{\mathrm{Mo}} + n_{\sum \mathrm{O}} \times M_{\mathrm{O}} + 2 \times n_{\mathrm{O,evap}} \times M_{\mathrm{H}}} \end{aligned} \tag{S14}$$

The following data from TGA analysis is used for the calculation:

180 °C: 95.5 wt.%

200 °C: 95.35 wt.%

550 °C: 85.46 wt.%

600 °C: 85.49 wt.%

Table S3. Calculated amount of detected water and its contribution of originating from the crystal (water + hydroxide) and reversibly bond water in NMO-H_2O-sheets, normalized to molybdenum content. Two different temperatures for both, after removal of reversibly bond water (550 °C and 600 °C) and after removal of reversibly bond and water originating from the crystal (180 °C and 200 °C) is chosen.

	Water evaporated	Water originating from the crystal	Reversibly bond water
20 °C – 550 °C	3.21		
20 °C – 180 °C		2.21	0.99
20 °C – 200 °C		2.18	1.03
20 °C – 600 °C	3.20		
20 °C – 180 °C		2.21	0.99
20 °C – 200 °C		2.17	1.03

The percental loss of weight after removal of all water compared to before the removal of crystal water and hydroxides for the two NMO-H_2O-sheets samples is (10.43 ± 0.07) % and (10.88 ± 0.12) % for the dried and not dried sample, respectively. This indicates that no significant amount of crystal water or hydroxides from the crystal were removed during the drying process also for this nanostructure.

In case the elemental concentration detected by IBA represents the material without reversibly bond water ($n_{\mathrm{RW}} = 0$), the following equation was utilized to calculate $n_{\mathrm{O}} + \frac{1}{2} n_{OH}$:

$$\frac{wt.\%_{\text{no CW and OH}}}{wt.\%_{\text{with CW and OH}}} = \frac{n_{\mathrm{Ni}} \times M_{\mathrm{Ni}} + n_{\mathrm{Mo}} \times M_{\mathrm{Mo}} + (n_{\sum \mathrm{O}} - (n_{\mathrm{O}} + \frac{1}{2} n_{OH})) \times M_{\mathrm{O}}}{n_{\mathrm{Ni}} \times M_{\mathrm{Ni}} + n_{\mathrm{Mo}} \times M_{\mathrm{Mo}} + n_{\sum \mathrm{O}} \times M_{\mathrm{O}} + (n_{\mathrm{O}} + \frac{1}{2} n_{OH}) \times 2 \times M_H} \quad \text{(S15)}$$

With $wt.\%_{\text{no CW and OH}}$ being the weight at 550°C and 600°C and $wt.\%_{\text{with CW and OH}}$ the weight at 180°C and 200°C.

Zero Field Cooled (ZFC) and Field Cooled (FC) measurement of NMO-H_2O nanostructures

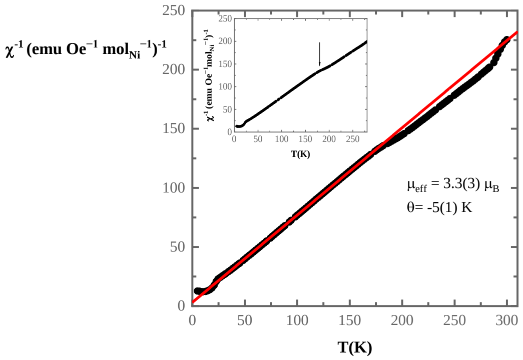

Figure S11. From **Figure 4a** derived inverse of the magnetic susceptibility χ^{-1} over temperature.

The inverse of the magnetic susceptibility χ^{-1} was plotted over the temperature. At high temperatures the magnetic susceptibility in the paramagnetic regime follows the characteristic $\frac{C}{T-\theta}$ behavior (with C as Curie constant, T as temperature and ϑ as Curie-Weiss temperature), known as the Curie-Weiss law. The linear fit in the range 25 K – 180 K led to an effective magnetic momentum derived from the C as μ_{eff} = 3.3 μ_B, with μ_B as Bohr magneton, and a Curie-Weiss temperature of ϑ = -5 K and, indicating antiferromagnetic behavior. A fit between 210 K to 280 K resulted in a slightly different magnetic moment μ_{eff} = 3.6 μ_B, which could originate from a Ni^{2+} in a tetrahedral ligand field, a magnetic anomaly, the presence of small impurities, or structural changes owing to the water coordination.

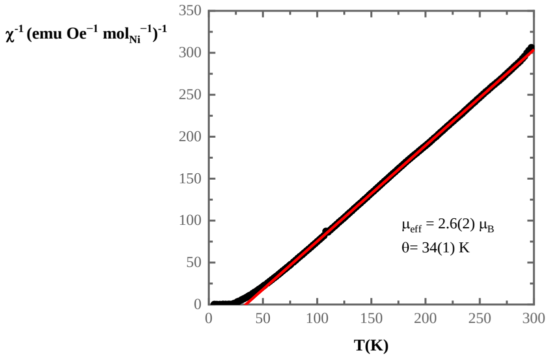

Figure S12. From **Figure 4b** derived inverse of the magnetic susceptibility χ^{-1} over temperature.

The inverse of the magnetic susceptibility χ^{-1} was plotted over the temperature. The linear fit in the range 25 K – 280 K led to an effective magnetic moment of μ_{eff} = 2.6 μ_B and a Curie-Weiss temperature of ϑ = 34 K, indicating ferromagnetic behavior, which likely agrees with the hysteresis loop at low temperature presented in the main work.

Powder X-Ray Diffraction (PXRD)

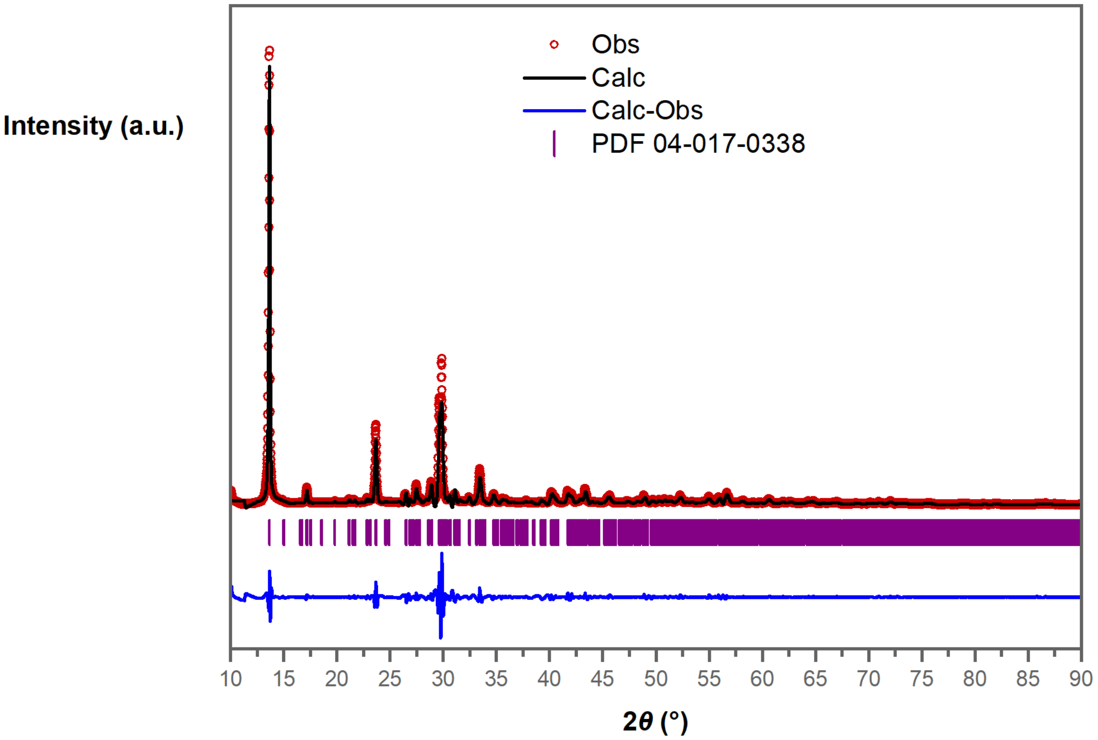

Figure S13. PXRD data for clarification with the observed data (red circles), the calculated data after refinement with the Le Bail methods (black line), the difference between calculated and observed intensity (blue line), and the angles at which the PDF 04-017-0338 reports reflexes.

Transmission Electron Microscopy (TEM) of NMO-H_2O nanostructures

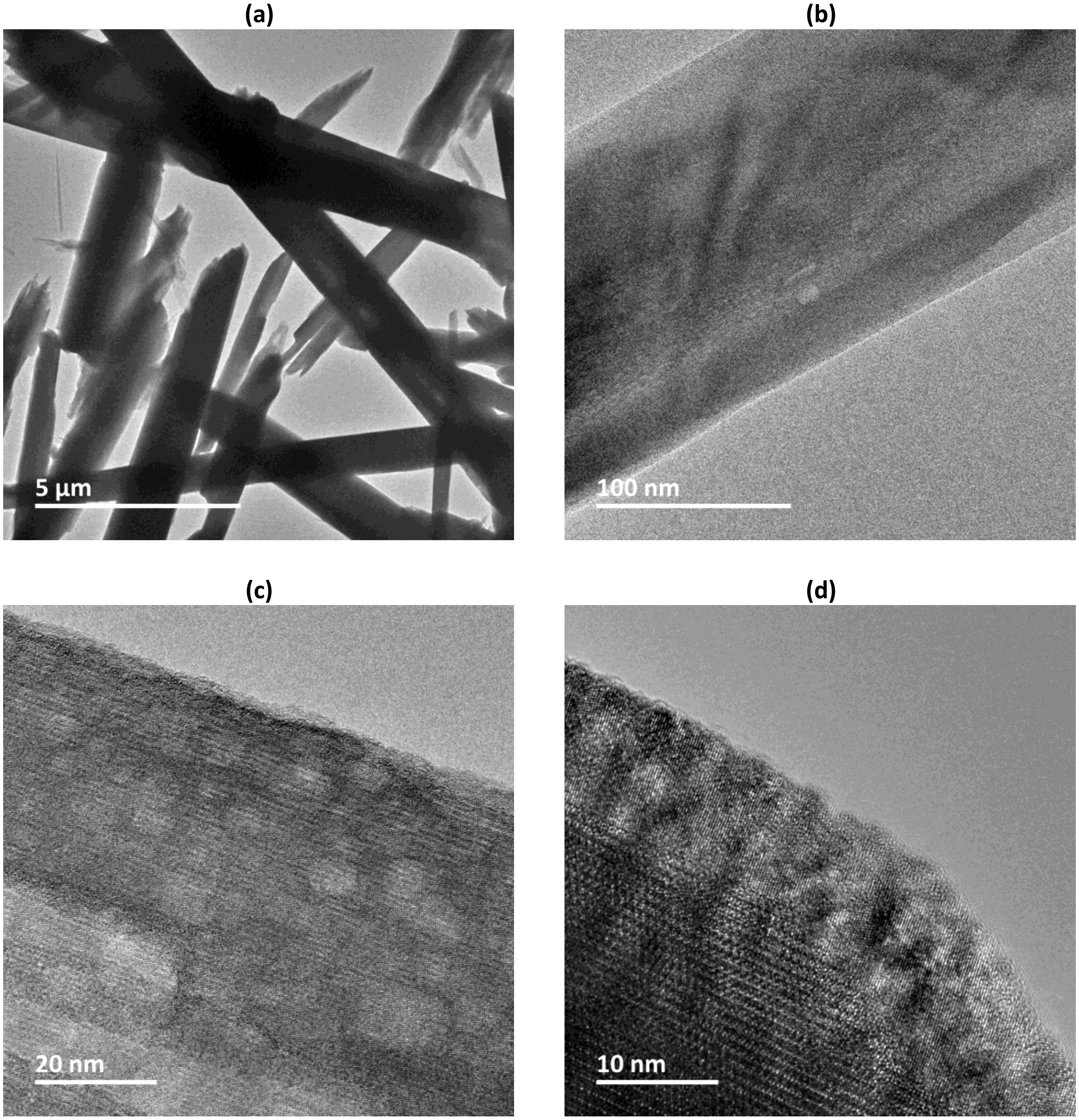

Figure S14. TEM analysis of NMO-H_2O-rods. **(a) – (d)** Overall morphology and lattice resolution TEM images of the rods. **(c)** and **(d)** indicate likely damages on the surface.

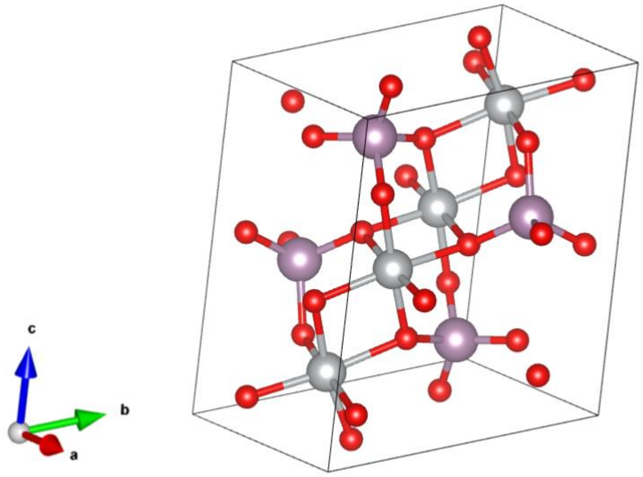

Figure S15. Illustration of the unit cell derived from 3D-electron diffraction with oxygen illustrated as red balls, nickel as grey balls and molybdenum as violet balls. The derived unit cell has the parameters a = 6.85000 Å, b = 6.99000 Å, c = 9.25000 Å, α = 76.3500 °, ß = 83.9200 °, and γ = 74.4600 °.

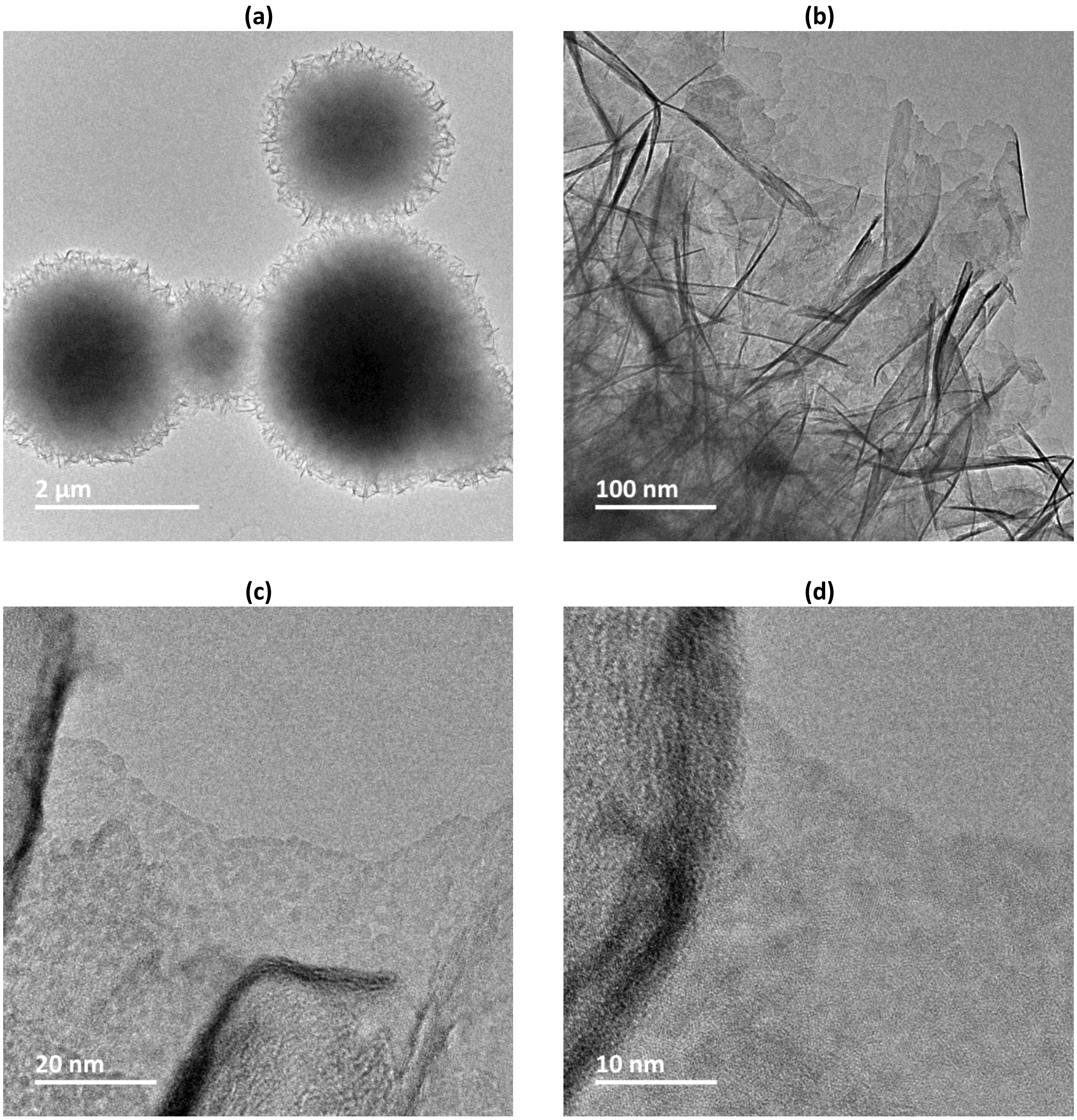

Figure S16. TEM analysis of NMO-H_2O-sheets. **(a)** – **(d)** TEM images of an assembly of sheets and sheets taken at different magnifications. **(b)** – **(d)** show the existence of small crystalline regions in a sheet.

Raman Spectroscopy of NMO-H_2O nanostructures

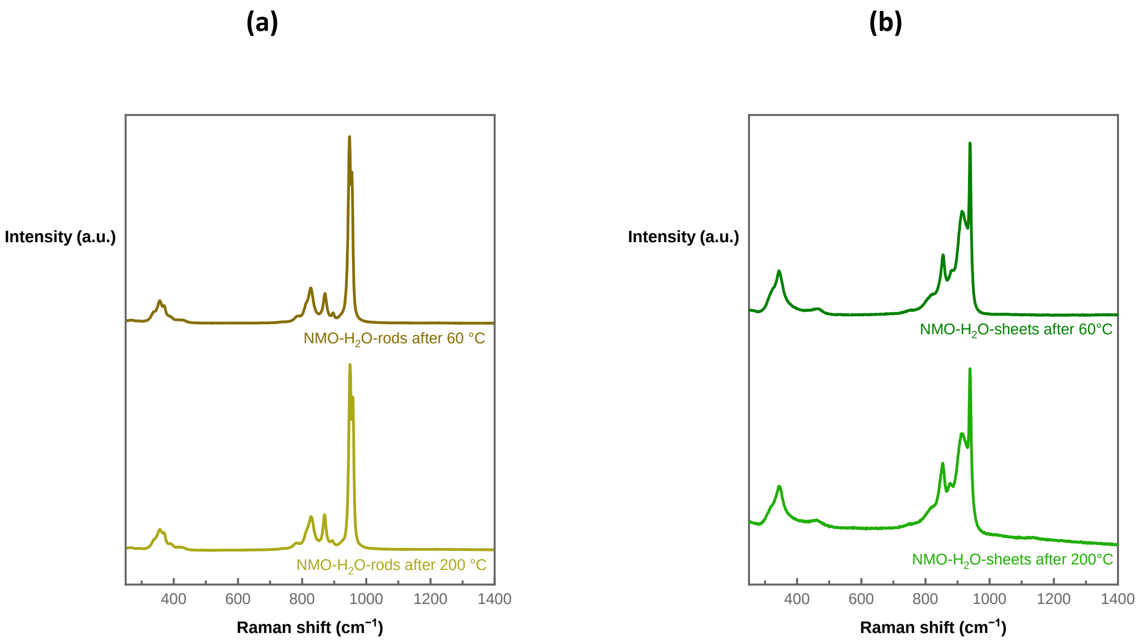

Figure S17. Raman spectroscopy with of the compounds before and after drying at 200 °C. **(a)** NMO-H_2O-rods spectra acquired after 60 °C with 1 s acquisition time, 10 % laser intensity and 5 acquisitions. The intensity is multiplied with 2.9 to reach similar intensities as the sample after 200 °C, which was acquired with 5 s acquisition time, 10 % laser intensity and 10 acquisitions. **(b)** NMO-H_2O-sheets spectra acquired after 60 °C and 200 °C with 5 s acquisition time, 10 % laser intensity and 10 acquisitions.

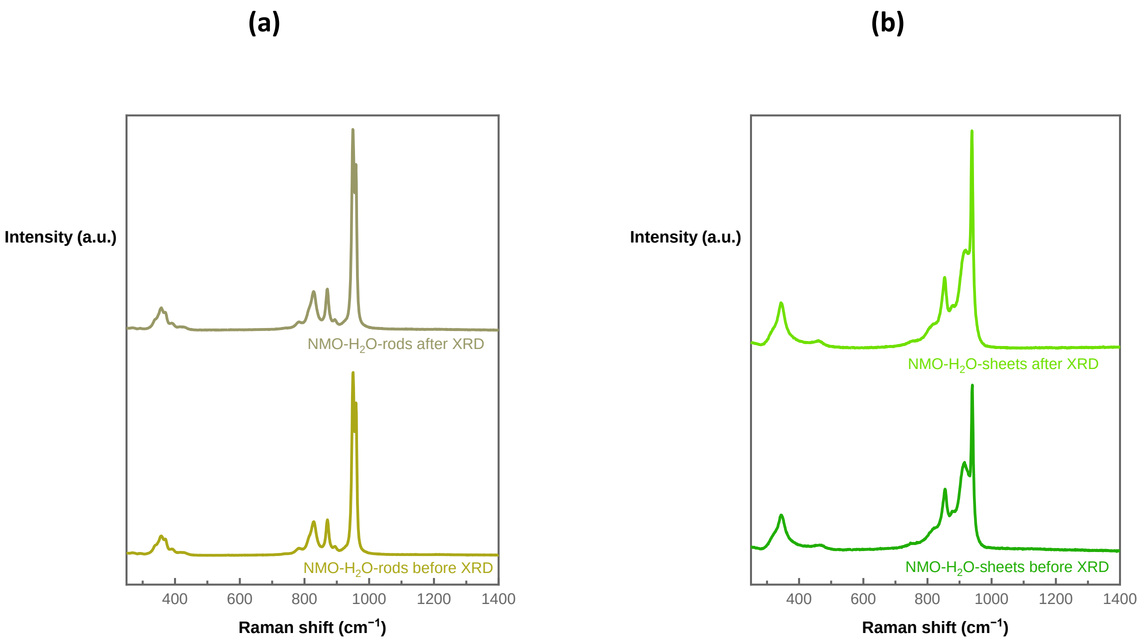

Figure S18. Raman spectroscopy on the NMO-H_2O samples before and after PXRD to confirm now structural change during the analysis. **(a)** NMO-H_2O-rods spectra with 10 and 5 s acquisition time for before and after PXRD, respectively, 5 % laser intensity and 5 acquisitions. **(b)** NMO-H_2O-sheets spectra with 5 s acquisition time, 10 % laser intensity and 10 acquisitions.

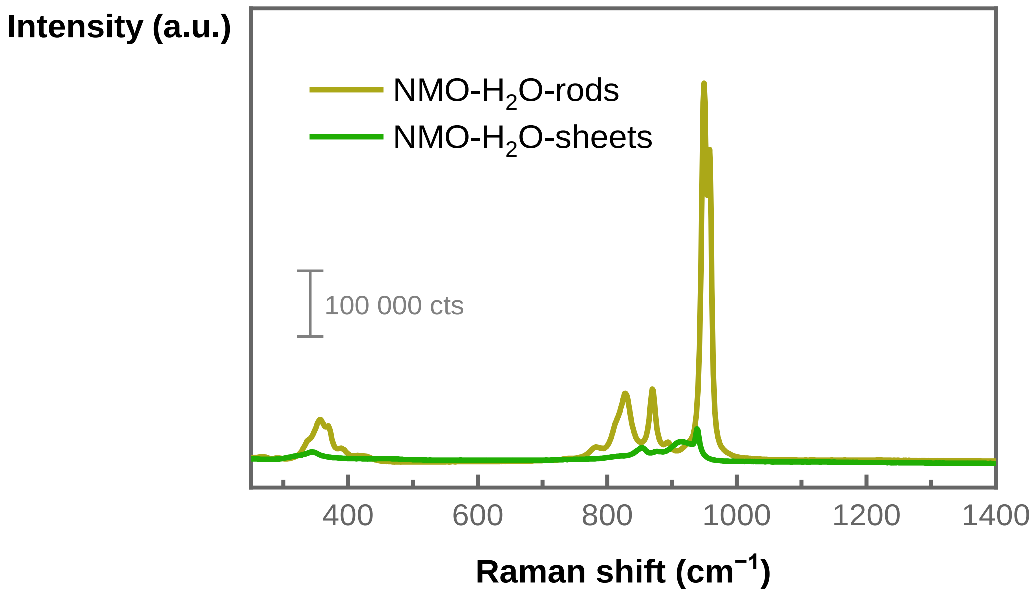

Figure S19. Raman spectra of the different NMO-H_2O nanostructures with identical acquisition conditions (5 s acquisition time, 10 % laser intensity and 10 acquisitions), showing the significant difference in peak intensities between the structures.

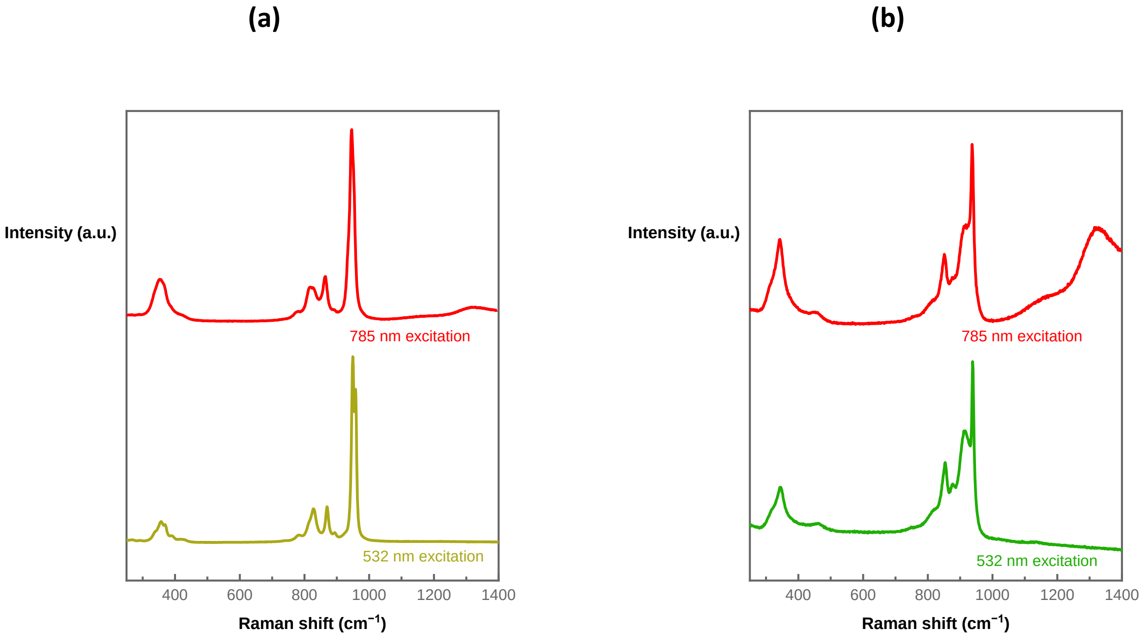

Figure S20. Raman spectra collected with 532 nm and 785 nm laser excitation to exclude resonance Raman effects in the 532 nm spectra. **(a)** NMO-H_2O-rods collected with 5 s acquisition time, 10 % laser intensity and 10 acquisitions. The intensity of the 785 nm spectra was multiplied by 4 to possess a comparable intensity as the 532 nm spectra. Slight differences like merging of peaks in the 785 nm spectra are attributed to the smaller grating used resulting in a decreased resolution. **(b)** NMO-H_2O-sheets collected with 5 s acquisition time, 10 % laser intensity and 10 acquisitions. The intensity of the 785 nm spectra was multiplied by 2.5 to possess a comparable intensity as the 532 nm spectra. The additional peaks for Raman shifts > 1000 cm^{-1} might originate from magnons or plasmons for both nanostructures.

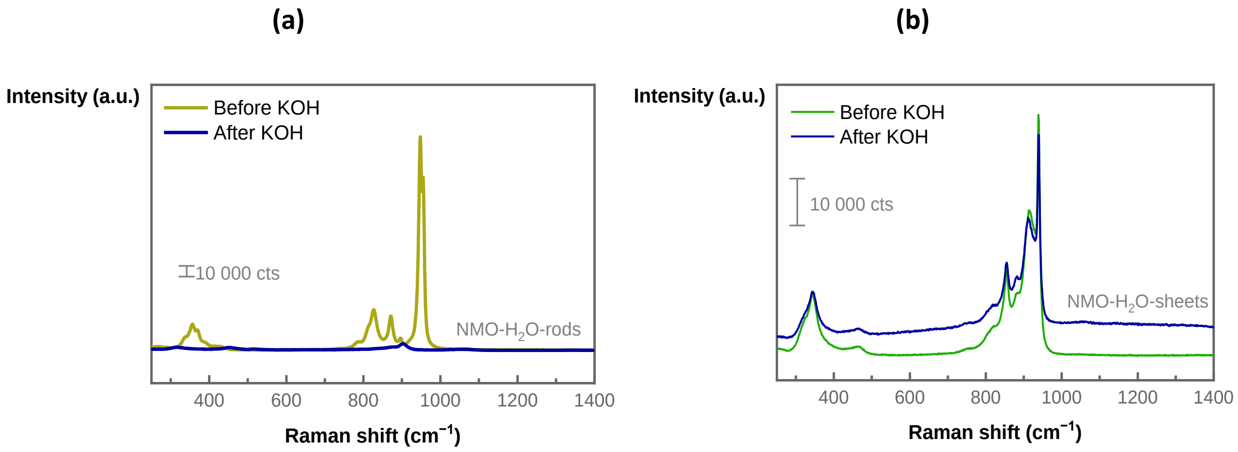

Figure S21. Selective molybdenum leaching from the NMO-H_2O-rods in 1 M KOH presents different chemical stabilities of the two compounds and no NMO-H_2O sheets in the NMO-H_2O-rods material. **(a)** NMO-H_2O-rods before (yellow) and after (blue) 6.5 hours in 1 M KOH. Spectra collected with 1 s acquisition time, 10 % laser intensity and 5 acquisitions before KOH and 5 s acquisition time, 10 % laser intensity and 10 acquisitions after KOH exposure. **(b)** NMO-H_2O-sheets before (green) and after (blue) 6 hours in 1 M KOH. Both spectra collected with 5 s acquisition time, 10 % laser intensity and 10 acquisitions.

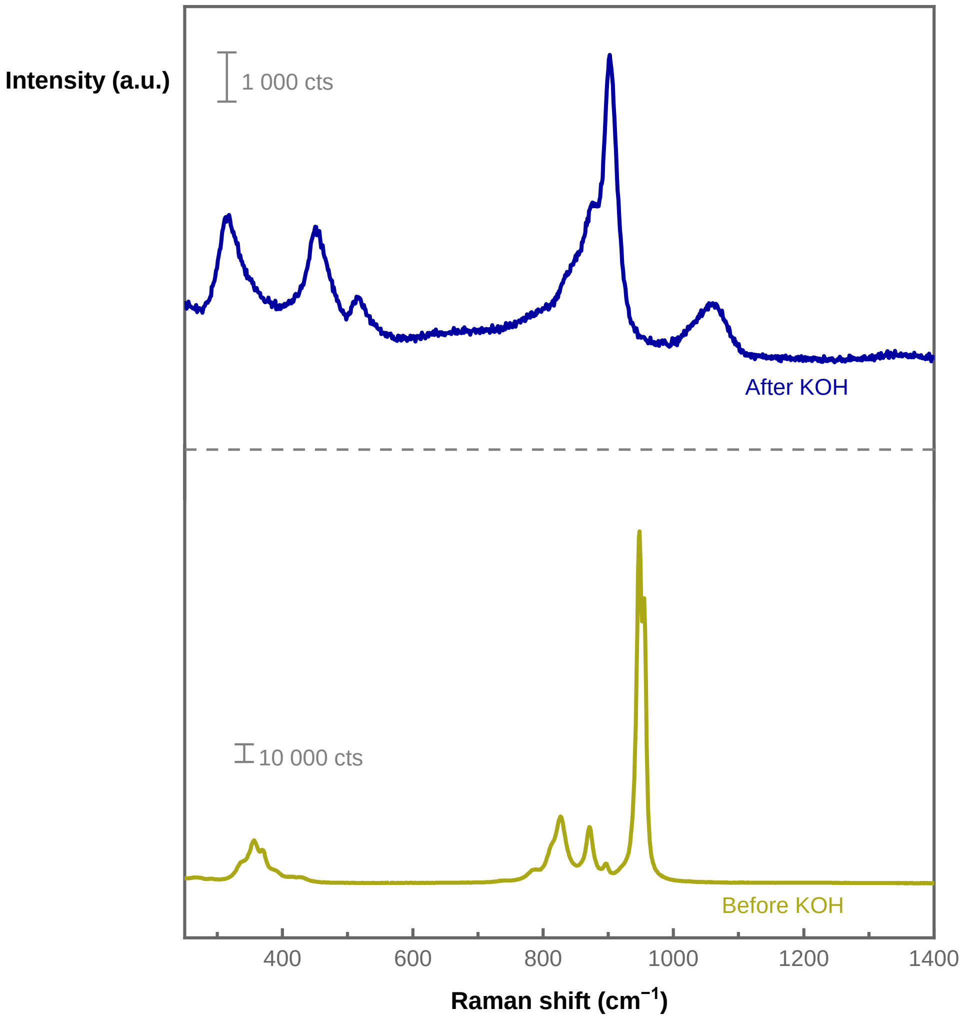

Figure S22. Raman spectra of NMO-H_2O-rods before (yellow) and after (blue) exposure to 1 M KOH. New peaks are detected at 1060 cm^{-1}, 902 cm^{-1}, 874 cm^{-1}, 517 cm^{-1}, 455 cm^{-1}, and 316 cm^{-1}.

Fourier Transform Infrared Spectroscopy (FTIR) of NMO-H_2O nanostructures

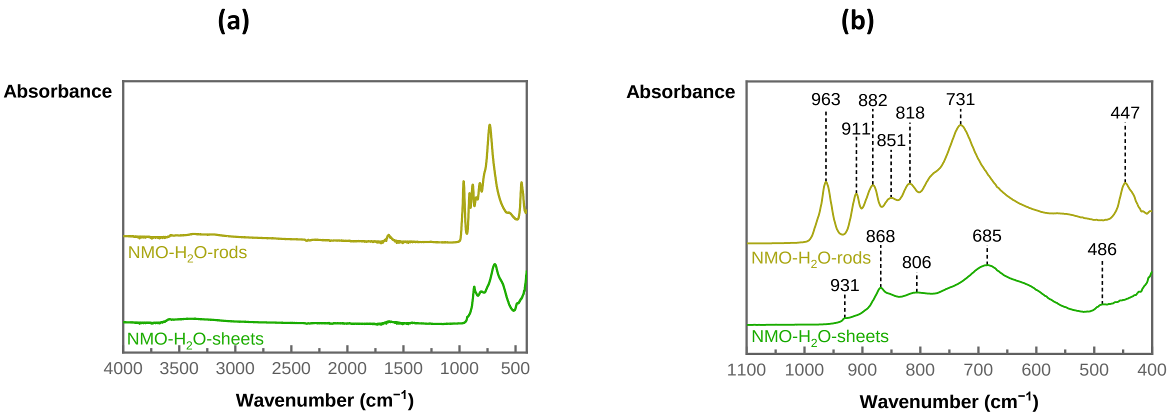

Figure S23. FTIR spectra of NMO-H_2O-rods and NMO-H_2O-sheets. **(a)** Long range with water vibrations between 3600 – 3000 cm^{-1} and at around 1630 cm^{-1}. **(b)** Fingerprint region.

Density-Functional Theory (DFT) Calculations

With DFT-calculations the PDF 04-017-0338 crystal structure was optimized (as explained in the computational section). The global optimization leads to the crystallographic data below as the ground state structure.

```
#======================================================================
# CRYSTAL DATA
#----------------------------------------------------------------------
data_VESTA_phase_1


_chemical_name_common                  'Ni Mo  O  H  O  H                    '
_cell_length_a                         6.787133
_cell_length_b                         6.900089
_cell_length_c                         9.239162
_cell_angle_alpha                      76.387207
_cell_angle_beta                       83.760468
_cell_angle_gamma                      73.390137
_cell_volume                           402.586103
_space_group_name_H-M_alt              'P 1'
_space_group_IT_number                 1


loop_
_space_group_symop_operation_xyz
   'x, y, z'


loop_
   _atom_site_label
   _atom_site_occupancy
   _atom_site_fract_x
   _atom_site_fract_y
```

_atom_site_fract_z

_atom_site_adp_type

_atom_site_U_iso_or_equiv

_atom_site_type_symbol

Ni1 1.0 0.762181 0.158646 0.042161 Uiso ? Ni

Ni2 1.0 0.147396 0.812537 -0.010976 Uiso ? Ni

Ni3 1.0 0.796903 0.338201 0.315936 Uiso ? Ni

Ni4 1.0 0.115511 0.630557 0.714950 Uiso ? Ni

Mo1 1.0 0.943770 0.787896 0.330702 Uiso ? Mo

Mo2 1.0 -0.036089 0.181215 0.701333 Uiso ? Mo

Mo3 1.0 0.706094 0.696108 -0.038240 Uiso ? Mo

Mo4 1.0 0.202934 0.276655 0.068918 Uiso ? Mo

O1 1.0 0.036248 0.757398 0.508153 Uiso ? O

O2 1.0 -0.123172 0.208863 0.523114 Uiso ? O

O3 1.0 0.170045 0.710003 0.213373 Uiso ? O

O4 1.0 0.734489 0.257896 0.817587 Uiso ? O

O5 1.0 0.440008 0.807588 -0.044478 Uiso ? O

O6 1.0 0.469304 0.165809 0.076864 Uiso ? O

O7 1.0 0.797507 0.689358 0.775054 Uiso ? O

O8 1.0 0.110571 0.293788 0.253039 Uiso ? O

O9 1.0 0.812804 0.062635 0.262735 Uiso ? O

O10 1.0 0.104888 0.908380 0.765118 Uiso ? O

O11 1.0 0.780178 0.623053 0.339996 Uiso ? O

O12 1.0 0.124727 0.347767 0.690317 Uiso ? O

O13 1.0 0.831527 0.856565 0.029685 Uiso ? O

O14 1.0 0.077704 0.115360 0.000713 Uiso ? O

O15 1.0 0.749029 0.438634 0.084476 Uiso ? O

O16 1.0 0.160726 0.533511 0.944301 Uiso ? O

O17 1.0 0.491203 0.369388 0.398074 Uiso ? O

O18	1.0	0.423090	0.569167	0.642059	Uiso ? O
H1	1.0	0.441891	0.501921	0.555366	Uiso ? H
H2	1.0	0.379623	0.445379	0.329743	Uiso ? H
H3	1.0	0.523950	0.471996	0.713722	Uiso ? H
H4	1.0	0.477209	0.225548	0.441084	Uiso ? H
O1	1.0	0.525728	0.971631	0.536279	Uiso ? O
H1	1.0	0.629774	0.917462	0.611735	Uiso ? H
H2	1.0	0.456324	0.861287	0.552058	Uiso ? H

The simulated XRD pattern was compared with the experimental gathered data.

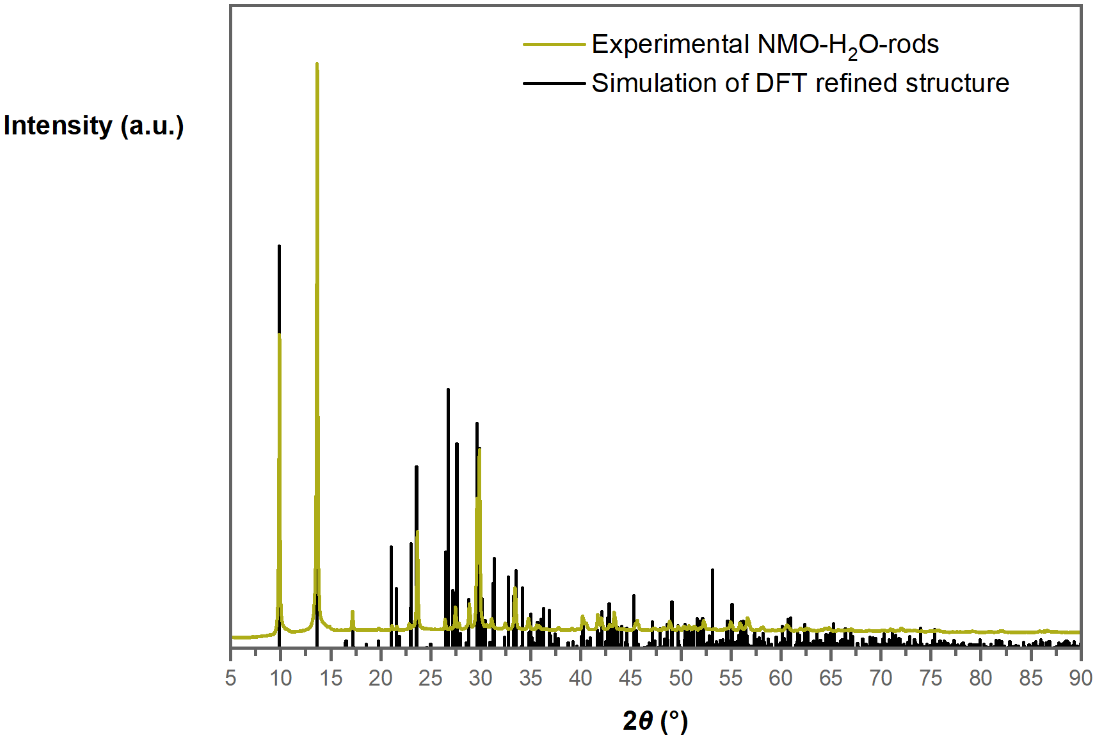

Figure S24. Comparison of experimental collected data of NMO-H_2O-rods (yellow) and the simulated PXRD pattern of the optimized crystal structure (black).

Bibliography

(1) Chen, J.; Zhao, G.; Chen, Y.; Rui, K.; Mao, H.; Dou, S. X.; Sun, W. Iron- Doped Nickel Molybdate with Enhanced Oxygen Evolution Kinetics. *Chemistry – A European Journal* **2019**, *25* (1), 280–284. https://doi.org/10.1002/chem.201803844.

(2) Fairley, N.; Fernandez, V.; Richard- Plouet, M.; Guillot-Deudon, C.; Walton, J.; Smith, E.; Flahaut, D.; Greiner, M.; Biesinger, M.; Tougaard, S.; Morgan, D.; Baltrusaitis, J. Systematic and Collaborative Approach to Problem Solving Using X-Ray Photoelectron Spectroscopy. *Applied Surface Science Advances* **2021**, *5*, 100112. https://doi.org/10.1016/j.apsadv.2021.100112.

(3) Schneider, C. A.; Rasband, W. S.; Eliceiri, K. W. NIH Image to ImageJ: 25 Years of Image Analysis. *Nat Methods* **2012**, *9* (7), 671–675. https://doi.org/10.1038/nmeth.2089.

(4) Maria Roslova; Stef Smeets; Bin Wang; Thomas Thersleff; Hongyi Xu; Xiaodong Zou. InsteaDMatic: Towards Cross-Platform Automated Continuous Rotation Electron Diffraction. *Journal of Applied Crystallography* **2020**, *53* (5), 1217–1224. https://doi.org/10.1107/S1600576720009590.

(5) Wan, W.; Sun, J.; Su, J.; Hovmöller, S.; Zou, X. Rotation Electron Diffraction (RED) Software. December 2, 2013. https://doi.org/10.5281/ZENODO.2545322.

(6) Kabsch, W. *XDS*. *Acta Crystallographica Section D Biological Crystallography* **2010**, *66* (2), 125–132. https://doi.org/10.1107/S0907444909047337.

(7) Hübschle, C. B.; Sheldrick, G. M.; Dittrich, B. ShelXle: A Qt Graphical User Interface for SHELXL. *Journal of Applied Crystallography* **2011**, *44* (6), 1281–1284. https://doi.org/10.1107/S0021889811043202.

(8) Sheldrick, G. M. SHELXT - Integrated Space-Group and Crystal-Structure Determination. *Acta Crystallographica Section A: Foundations of Crystallography* **2015**, *71* (1), 3–8. https://doi.org/10.1107/S2053273314026370.

(9) Momma, K.; Izumi, F. VESTA 3 for Three-Dimensional Visualization of Crystal, Volumetric and Morphology Data. *J Appl Cryst* **2011**, *44* (6), 1272–1276. https://doi.org/10.1107/S0021889811038970.

(10) Mayer, M. SIMNRA, a Simulation Program for the Analysis of NRA, RBS and ERDA. In *AIP Conference Proceedings*; AIP: Denton, Texas (USA), 1999; pp 541–544. https://doi.org/10.1063/1.59188.

(11) Ström, P.; Petersson, P.; Rubel, M.; Possnert, G. A Combined Segmented Anode Gas Ionization Chamber and Time-of-Flight Detector for Heavy Ion Elastic Recoil Detection Analysis. *Review of Scientific Instruments* **2016**, *87* (10), 103303. https://doi.org/10.1063/1.4963709.

(12) Arstila, K.; Julin, J.; Laitinen, M. I.; Aalto, J.; Konu, T.; Kärkkäinen, S.; Rahkonen, S.; Raunio, M.; Itkonen, J.; Santanen, J. P.; Tuovinen, T.; Sajavaara, T. Potku - New Analysis Software for Heavy Ion Elastic Recoil Detection Analysis. *Nuclear Instruments and Methods in Physics Research, Section B: Beam Interactions with Materials and Atoms* **2014**, *331*, 34–41. https://doi.org/10.1016/j.nimb.2014.02.016.

(13) Adel, M. E.; Amir, O.; Kalish, R.; Feldman, L. C. Ion-Beam-Induced Hydrogen Release from a-C:H: A Bulk Molecular Recombination Model. *Journal of Applied Physics* **1989**, *66* (7), 3248–3251. https://doi.org/10.1063/1.344116.

(14) Ström, P. Material Characterization for Magnetically Confined Fusion: Surface Analysis and Method Development, KTH Royal Institute of Technology, 2019.
(15) *Wolfram|Alpha: Making the world's knowledge computable*. https://www.wolframalpha.com (accessed 2023-06-15).
